# Clinical and Preclinical Systematic Review of *Panax ginseng* C. A. Mey and Its Compounds for Fatigue

**DOI:** 10.3389/fphar.2020.01031

**Published:** 2020-07-17

**Authors:** Ting-Yu Jin, Pei-Qing Rong, Hai-Yong Liang, Pei-Pei Zhang, Guo-Qing Zheng, Yan Lin

**Affiliations:** Department of Neurology, The Second Affiliated Hospital and Yuying Children's Hospital of Wenzhou Medical University, Wenzhou, China

**Keywords:** ginseng, fatigue, randomized controlled trials, animal studies, systematic review

## Abstract

**Background:**

Fatigue, as a complex, multidimensional symptom, is associated with many physical illnesses. *Panax ginseng* C. A. Mey (PG) is an important herbal drug which has been used for benefiting Qi for thousand years. *Panax ginseng* C. A. Mey and its compounds (PGC) possess various pharmacological activities, including anti-fatigue. Here, we conducted a systematic review of both randomized clinical trials (RCTs) and preclinical animal studies to investigate the efficacy and safety of PGC for fatigue.

**Methods:**

Electronic searches were performed in 7 databases from the time of each database's inception to August 2019. The methodological quality of RCTs was assessed using 7-item checklist recommended by Cochrane Collaboration or by the CAMARADES 10-item quality checklist. All the data were analyzed using Rev-Man 5.3 and Stata SE software.

**Results:**

Eight eligible RCTs and 30 animal studies were identified. The risk of bias scores in RCTs ranged from 4/7 to 7/7, and of animal studies varied from 4/10 to 7/10. Meta-analyses showed that PGC was superior to placebo according to their respective fatigue scales, heart rate recovery, and clinical effect (P < 0.05). There were a similar number of adverse effects between PGC and placebo group (P > 0.05). Meta-analyses showed that PGC can significantly decrease level of blood lactate, blood urea nitrogen, creatine kinase, malondialdehyde, and lactic dehydrogenase in serum, level of malondialdehyde in liver and level of gamma-aminobutyric acid, 5-hydroxytryptamine in brain tissue, and increase swimming time, level of glutathione peroxidase, glucose, superoxide dismutase in serum, level of glycogen and activity of superoxide dismutase, glutathione peroxidase, and catalase in skeletal muscle, level of hepatic glycogen in liver and level of dopamine, acetylcholine in brain tissue, compared with control (P < 0.05). Meta-analyses showed no significant difference in animal body weight between PGC and control (P > 0.05).

**Conclusion:**

The present findings supported, to a certain degree, that PGC can be recommended for routine use in fatigue. The possible mechanism of PGC resists fatigue, mainly through antioxidant stress, regulating carbohydrate metabolism, delaying the accumulation of metabolites, promoting mitochondrial function, neuroprotection, antiapoptosis, and regulating neurotransmitter disorder in central nervous system.

## Introduction

### Description of the Condition

Fatigue is a condition of lacking the energy and motivation in responding to physical activity, emotional stress, boredom or insufficient sleep (Bach et al., 2016). It is a complex, multidimensional symptom that is prevalent in the general population (Jason et al., 2010). The cause of fatigue is unknown. The severity of fatigue varies greatly among individuals. Although fatigue does not lead to death, it has negative impacts on many areas of daily life (Arring et al., 2018). “Feeling weak all over for much of the time” was regarded as one of the most important symptoms (Lewis and Wessely, 1992). Most physical illnesses are associated with fatigue, such as many chronic diseases, namely anaemia, emphysema, asthma, and arthritis (Chen, 1986). Fatigue is a clinical challenge because its study of etiology, risk factors, and pathophysiology are still at an early stage. The goal of treatment is to treat symptoms and improve outcomes rather than to provide clear treatment (Alraek et al., 2011).

### Description of the Intervention

Different interventions have been used in treating fatigue (Whiting et al., 2001). Nowadays, treatments commonly focus on muscle pain, sleep disorders, and emotional symptoms. Cognitive Behavior Therapy (CBT), various forms of exercise, as well as enhancement of coping ability, are standard treatment options. In addition, caregiver prescribed or self-administered medication are still common (Jones et al., 2007). It is indicated that no universal western medicine treatment can be recommended (Collatz et al., 2016). Interventions which include CBT and graded exercise therapy have shown promising results (Whiting et al., 2001). However, patients seem to be skeptical about CBT, who claimed that CBT and graded exercise for fatigue were neither effective nor safe (Twisk and Corsius, 2018). Recently, various forms of complementary and alternative medicine (CAM) have been widely used in fatigue such as herbal medicine, cheirapsis, balanced nutrition, and acupuncture (Jones et al., 2007). In particular, *Panax ginseng* C.A. Mey (PG) has been a rising utilization in treating fatigue in Asia and elsewhere around world. Based on traditional Chinese medicine and herbal philosophy, PG is considered as an adaptation to help restore body balance (Arring et al., 2018). *Panax ginseng* C.A. Mey is believed to improve overall quality of life (QoL), including energy and vitality, particularly during times of fatigue or stress (Yennurajalingam et al., 2017).

### How the Intervention Might Work

*Panax ginseng* C.A. Mey has direct effects on the central nervous system (CNS), including cognition, sleep disorders, depression, pain, and the ability to regulate inflammatory cytokines (Yennurajalingam et al., 2017). To date, numerous active compounds have been identified such as ginsenosides, ginseng polysaccharides, and ginseng protein. Ginsenosides, the most important ingredients of ginseng, have been proved with various pharmacological activities such as anti-fatigue, anti-oxidation, neuroprotection, anti-inflammation, and anti-diabetes. Ginsenoside Rg3 (Rg3) is one of the most abundant ginsenosides. It may improve exercise performance and increase fatigue resistance by enhancing deacetylase activity of silent information regulator of transcription 1 (SIRT1) and inhibiting the transcriptional activity of p53 (Yang et al., 2018). Ginseng polysaccharides have anti-fatigue activity probably by mobilizing triglyceride (TG) or fat during exercise, or by changing the activities oflactic dehydrogenase (LDH), malondialdehyde (MDA) and glutathione peroxidase (GPH-Px) to avoid lipid oxidation and protect corpuscular membrane (Zheng et al., 2017). Ginseng proteins could resist fatigue through retarding the accumulation of blood lactate (BLA) and blood urea nitrogen (BUN), enhancing hepatic glycogen levels, and improving the ability of antioxidant enzymes (Qi et al., 2014).

### Why It Is Important to Do This Review

*Panax ginseng* C.A. Mey is one of the most widely used plant products worldwide which has been used in oriental countries for thousands of years (Zheng et al., 2017). Based on a comprehensive collection of clinical trials, systematic review can perform comprehensive analysis and statistical processing on qualified studies to form relatively reliable results, which can guide clinical decision-making. In addition, systematic review can solve the following clinical problems: research on the effectiveness of treatment, evaluation of diagnostic methods, prognosis estimation, analysis of the cost and benefit of treatment. Up to now, at least 2 systematic reviews have been conducted to evaluate efficacy and safety of *Panax ginseng* C. A. Mey and its compounds (PGC) for fatigue (Bach et al., 2016; Arring et al., 2018). However, the results of these reviews are inconclusive because of methodological flaws in their included primary studies. Cochrane group have developed an extensive set of guide lines for systematic reviews. These “not-so-good” studies were excluded with a strict process (Xie et al., 2013). In addition, the efficacy and mechanisms of PGC in fatigue animal models have not been systematically evaluated yet. Systematic review of animal researches is indispensable in the process of drug development and elucidation of the physiological and pathological mechanisms (Zheng et al., 2018). Preclinical research is the key to convert preclinical data into clinical data. In addition, systematic review of animal research is a more economic and ethical approach, which can integrate preclinical evidence, help reduce unnecessary sacrifice of laboratory animals, and prevent ineffective or less informative research (Zhou et al., 2019). As we all know, there is a gap between clinical research and clinical practice. More communication is needed between animal researchers and clinical researchers. Systematic review of animal experiments can lead to better collaboration between research groups and encourage the use of iterative methods to improve the relevance of animal models to clinical trial design. If the model cannot well simulate the clinical situation, it can be adjusted accordingly. In addition, as in human research, systematic review helps to identify and improve behavioral and reporting deficiencies in animal research Perel et al., 2007. Systematic review can effectively integrate preclinical comprehensive evidence and guide potential clinical translation. Thus, the aim of present study was to systematically summarize and critically evaluate the data from randomized control trials (RCTs) and animal studies of PGC for fatigue.

## Methods

### Search Strategy

This study followed the PRISMA statement (Stewart et al., 2015). EMBASE, PubMed, Cochrane Library, China National Knowledge Infrastructure (CNKI), VIP database (VIP), China Biology Medicine Database (CBM) and Wangfang database were electronically searched from their inception to August 2019. The following keywords were used: “fatigue OR Lassitude lethargy OR exhaustion OR weariness OR tiredness” and “panax OR Ginseng OR renshen” in Chinese or in English. All searches were limited to animal studies and clinical trials.

### Eligibility Criteria

The prespecified inclusion criteria of RCTs listed below: (1) RCTs that evaluated the effectiveness and safety of PGC for fatigue; (2) the Cochrane risk of bias (ROB) tool met at least 4 out of the 7 domains; (3) Subjects had chronic fatigue syndrome (CFS) or healthy adults after exercise; Subjects were classified as CFS-like according to Evaluation and Classification of Unexplained Chronic Fatigue (ECUCF) (Fukuda et al., 1994); (4) PGC as monotherapy was used as an intervention in the treatment group, and interventions for control group were placebo or vehicle treatment; (5) The primary outcome measures were scales of fatigue and/or objective evaluation criteria (e.g. physical performance, biochemical parameters). The secondary outcome measures were clinical effect according to fatigue scales and adverse events. The exclusion criteria were prespecified as follows: (1) fatigue caused by a medical condition, or withdrawal from medicines or substance; (2) duplicate publications and no available data.

The inclusion criteria of animal studies were prespecified as follows: (1) PGC for fatigue animal models was established by forced movement; (2) The interventions of treatment group were PGC at any dose and control group were nonfunctional liquid (normal saline) or no treatment; and (3) The primary outcome measures were forced movement time and/or serum biochemical parameters and/or skeletal muscle biochemical parameters and/or liver biochemical parameters and/or brain tissue biochemical parameters. The secondary outcome measures were body weight, organ index (organ weight/body weight) and possible mechanisms of PGC for anti-fatigue. The exclusion criteria were predefined as follows: (1) not fatigue model; (2) combined use of other drugs; and (3) no available data, duplicate publications, and lack of control group.

### Data Extraction

Two independent researchers extracted the details from the included RCTs and animal studies according to two standardized data extraction forms, respectively. There are many manners for including outcomes, such as peak time point, last time point, and same time point. There is undeniable that any manner will lead to bias. In order to minimize bias, inclusion criteria were prespecified as follows: The result of the peak time point was included when the data were expressed at different times. If meta-analysis data were lost or expressed graphically, we would try to contact the author for more information. When no response was received, we used digital ruler software or exclusion software to measure data from charts. If the data in the primary RCT were missing or merely illustrated graphically, an effort was launched to obtain further information through contacting the authors. If failed, the digital ruler software was used for measuring data from the graphs or excluded.

### Quality Assessment

The methodological quality of the included RCTs was evaluated by using the Cochrane Collaboration's tool. The RoB of the included animal studies was assessed using 10-item quality checklist of the Collaborative Approach to Meta-Analysis and Review of Animal Data from Experimental Studies (CAMARADES) with minor modification. Divergences were well settled by correspondence author (GZ).

### Statistical Analysis

Analysis was conducted with RevMan 5.3 and Stata SE software. Continuous outcomes were presented as mean difference (MD) or standardized mean difference (SMD) with 95% confidence interval (CI). Dichotomous outcomes were presented risk ratio (RR) or odds ratio (OR) with 95% CI. Probability values P <0.05 were considered significant. In order to estimate heterogeneity across studies, we used I^2^ - statistic test. An I^2^ value greater than 50% was considered as having substantial heterogeneity. When substantial heterogeneity was not observed, the fixed-effects model was reported. On the contrary, the random-effects model was reported. Simultaneously, considering the differences in subjects, interventions, and treatments, we used the Z-test for subgroup analysis. P <0.05 was considered to be statistically significant. If an outcome contained more than 10 RCTs, funnel plots, and Egger's test were used to examine publication bias.

## Results

### Study Selection

**Figure 1:**
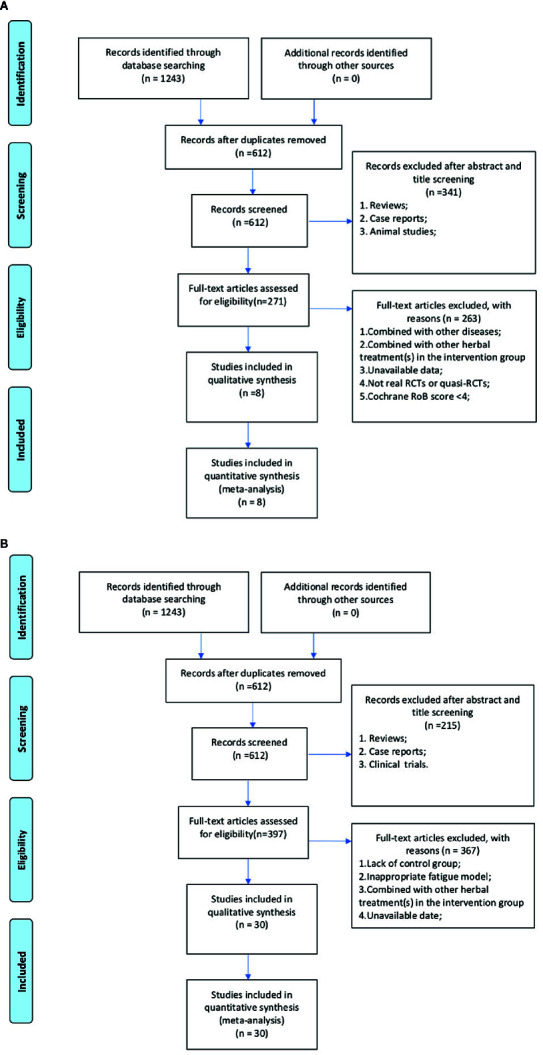
**(A)** Flow diagram of randomized control trials selection process. **(B)** Flow diagram of animal experiments selection process.

A total of 1331 relevant literatures were retrieved from the database, of which 667 were considered duplicates. Of the remaining 664 articles, qualified RCTs and animal experiments should be selected separately. For RCTs, 362 articles were eliminated because of reviews, case report, or animal studies. After scanning the remaining 302 full-text articles, 294 studies were excluded by reasons that they were (1) combined with other disease; (2) combined with other herbal treatment(s) in the intervention group; (3) no data available; (4) not real RCTs or quasi-RCTs; or (5) with the less than 4 domains “yes” according to the Cochrane RoB tool. Eventually, 8 RCTs were selected (**Figure 1A**). For animal studies, 243 studies including clinical trials, case reports or review articles were excluded. Through full-text evaluation of the remaining 421 studies, 391 were excluded for at least one of the following reasons: (1) lack of control group; (2) inappropriate fatigue model; (3) combined with other herbal treatment(s) in the intervention group; (4) unavailable data. Ultimately, 30 animal studies were included (**Figure 1B**).

### Study Characteristics

#### Randomized Control Trials

All involved studies were published in English. All 8 studies (Gal et al., 1996; Engels et al., 1996; Engels et al., 2001; Engels et al., 2003; Hartz et al., 2004; Hyeong-Geug et al., 2013; Kim et al., 2016; Lee et al., 2016) were RCTs, which involved a total of 678 participants, and the sample size ranged from 19 to 218. Five RCTs (Gal et al., 1996; Hartz et al., 2004; Hyeong-Geug et al., 2013; Kim et al., 2016; Lee et al., 2016) involved 615 participants with CFS. The diagnostic criteria of patients with CFS were based on ECUCF. The rest of 3 RCTs (Engels et al., 1996; Engels et al., 2001; Engels et al., 2003) involved 63 healthy adults after exercise. They were published from 1996 to 2016 and conducted in America (n = 4) (Engels et al., 1996; Engels et al., 2001; Engels et al., 2003; Hartz et al., 2004), South Korea (n = 3) (Hyeong-Geug et al., 2013; Kim et al., 2016; Lee et al., 2016), and France (n = 1) (Gal et al., 1996). The duration of treatment ranged from 4 to 8 weeks. They used PGC as intervention of experimental group. The placebo-control was used in all 8 studies. Five studies (Engels et al., 1996; Engels et al., 2001; Engels et al., 2003; Hyeong-Geug et al., 2013; Kim et al., 2016) used objective evaluation criteria, including metabolic response and physical performance as outcome measure. Five studies (Gal et al., 1996; Hartz et al., 2004; Hyeong-Geug et al., 2013; Kim et al., 2016; Lee et al., 2016) used self-reported fatigue measures, including Rand Vitality Index (RVI), Mood and Anxiety Symptom Questionnaire (MASQ), fatigue duration, checklist individual strength (CIS), Numeric Scale (NRS), Visual Analogue Scale (VAS), Visual Analogue Fatigue Scale (VAFS), Revised Piper Fatigue Scale (RPFS), Short-Form Health Survey (SF-36), Fatigue score, and clinical effect as outcome measure. The general characteristics of the 8 included articles were illustrated in **Tables 1** and **2**.

**Table 1:** Characteristics of the included randomized control trials.

First Authors; year	Study design	Gender (male/female); mean/range age(years)		Interventions		Course of treatment	Outcome measure	Intergroup differences
		Trail	Control	Trail	Control			
Engels et al., 1996	RCT	10 21–35	9 21–35	G115 (200 mg qd) po	placebo	8 weeks	blood lactic acid oxygen uptake RER VE heart rate	P > 0.05 P > 0.05 P > 0.05 P > 0.05 P > 0.05
Engels et al., 2001	RCT	9 27.1 ± 7.0	10 23.6 ± 5.4	G115 (400 mg qd) po	placebo	8 weeks	peak anaerobic power output mean anaerobic power output rate of fatigue immediate postexercise recovery heart rates	P > 0.05 P > 0.05 P > 0.05 P > 0.05 P < 0.05
Engels et al., 2003	RCT	15 26.3 ± 1.9	10 26.1 ± 1.7	G115 (400 mg qd) po	placebo	8 weeks	Peak power output mean power output Heart rate recovery	P < 0.01 P < 0.01 P > 0.05
Hartz et al., 2004	RCT	30/6 21–34 0 35–49 18 50–65 18	31/9 21–34 1 35–49 22 50–65 7	extract of PG (800 mg bid) po	placebo	2 months	Fatigue severity RVI MASQ Fatigue duration Adverse event	P < 0.05 P < 0.05 P < 0.05 P < 0.05 P > 0.05
Kim et al., 2016	RCT	23/49 < 29 27 30–39 25 40–49 16 > 50 4	15/62 < 29 23 30–39 36 40–49 13 > 50 3	URSA (1# 50 mg bid) po	placebo	4 weeks	CIS score ALT AST γ‐GT Adverse event	P > 0.05 P < 0.05 P > 0.05 P > 0.05 P > 0.05
Hyeong-Geug et al., 2013	RCT	21/69 39.5	6/24 39.5	PG (1, 2 g qd) po	placebo	4 weeks	NRS VAS ROS MDA GSH GSH-Rd Adverse event	P > 0.05 P < 0.05 P < 0.05 P < 0.05 P < 0.05 P < 0.05 P > 0.05
Lee et al., 2016	RCT	5/21 60.1 ± 4.44	7/19 62.1 ± 5.18	EMGE (500 mg bid) po	placebo	4 weeks	VAFS RPFS SF-36 Adverse event	P < 0.01 P > 0.05 P > 0.05 P > 0.05
Gal et al., 1996	RCT	34/75 37.6	34/75 38.8	Pharmaton Capsule (1# bid) po	placebo	42 days	Fatigue score Adverse event	P=0.019 P > 0.05

ALT, alanine aminotransferase; AST, aspartate aminotransferase; CIS, checklist individual strength; GSH, glutathione; GSH-Rd, glutathione reductase; G115, standardized P. ginseng C.A. Mey concentrate;MASQ, Mood and Anxiety Symptom Questionnaire; MDA, malondialdehyde; NRS, self-rating numeric scale; PG, P. ginseng C.A. Mey; RER, respiratory exchange ratio; ROS, reactive oxygen species; RPFS, Revised Piper Fatigue Scale; RVI, Rand Vitality Index; SF-36, Short-Form Health Survey; VAS, visual analog scale; VAFS, visual analog fatigue scale; VE, minute ventilation.

**Table 2:** Statement of the characteristic of *Panax ginseng* C. A. Mey and its compounds(a).

First Authors; year	Drugs;	Approach to achieving	Chemical analysis	Composition
Engels et al., 1996	G115	Pharmaten Ltd., Lugano, Switzerland	included-HPLC	Contain 4% ginsenosides(Rgl 0.548%, Re 0.352%, Rf 0.270%, Rg2 0.065%, Rbl 1.338%, Rc 0.714%, Rb2 0.567%, Rd 0.286%) HPLC data from F. Soldati and Sticher, 1980.
Engels et al., 2001	G115	Pharmaten Ltd., Lugano, Switzerland	included-HPLC	Contain 4% ginsenosides(Rgl 0.548%, Re 0.352%, Rf 0.270%, Rg2 0.065%, Rbl 1.338%, Rc 0.714%, Rb2 0.567%, Rd 0.286%) HPLC data from F. Soldati and Sticher, 1980.
Engels et al., 2003	G115	Pharmaten Ltd., Lugano, Switzerland	included-HPLC	Contain 4% ginsenosides(Rgl 0.548%, Re 0.352%, Rf 0.270%, Rg2 0.065%, Rbl 1.338%, Rc 0.714%, Rb2 0.567%, Rd 0.286%) HPLC data from F. Soldati and Sticher, 1980.
Hartz et al., 2004	extract ofPG	Frontier Herbs of Norway, IA., Norway	included-HPLC	Contain 0.112%eleutheroside B and eleutheroside E
Kim et al., 2016	URSA Complex (contain dried PG extract);	not reported	included-HPLC	Contain dried ginseng extracts 50 mg
Hyeong-Geug et al., 2013	bespoke 20% ethanol extract of PG;	Guryoung Pharmaceutical Company, Ltd., Cheorwon, South Korea	included-HPLC	Contain 2% ginsenosides (Rb3 0.633%, Rb1 0.514%, Rb2 0.36%, Rc 0.261%, Rg3 0.108%, Rd 0.043%, Rh2 0.0002%) HPLC data from Hyeong-Geug et al., 2013.
Lee et al., 2016	Enzyme-modified ginseng extract (EMGE);	not reported	included-HPLC	Rg1, Rh1, Rb1, Rg3(S), Rg3(R), Compound K, Rh2(S), Rh2(R);(similar to G115 by HPLC) HPLC data from Lee et al., 2016.
Gal et al., 1996	Pharmaton Capsule (contain G115);	Pharmaten Ltd., Lugano, Switzerland	included-HPLC	Contain 4% ginsenosides (Rgl 0.548%, Re 0.352%, Rf 0.270%, Rg2 0.065%, Rbl 1.338%, Rc 0.714%, Rb2 0.567%, Rd 0.286%) HPLC data from F. Soldati and Sticher, 1980.

G115, standardized P. ginseng C.A. Mey concentrate; HPLC: high-performance liquid chromatography; PG, Panaxginseng C.A. Mey.

#### Animal Studies

Nine studies (Choi et al., 2011; Hwang et al., 2014; Qi et al., 2014; Wang et al., 2014; Oh et al., 2015; Bao et al., 2016; Ma et al., 2017; Zheng et al., 2017; Delgado et al., 2019) were published in English and 21 studies (Feng et al., 2009a; Feng et al., 2009b; Feng et al., 2009c; Li et al., 2009; Pan et al., 2009; Feng et al., 2010a; Feng et al., 2010b; Feng et al., 2010c; Pan et al., 2010a; Pan et al., 2010b; Xu et al., 2010; Chen and Li, 2011; Gao et al., 2011; Xu et al., 2011; Song et al., 2013; Zhao et al., 2014; Liu et al., 2015; Wang et al., 2015; Liu, 2016; Shi et al., 2016; Yao, 2016) were published in Chinese between 2005 and 2019. All 30 studies involved a total of 2249 animals. Twelve studies (Feng et al., 2009a; Feng et al., 2009b; Feng et al., 2009c; Pan et al., 2009; Feng et al., 2010a; Feng et al., 2010b; Feng et al., 2010c; Pan et al., 2010a; Pan et al., 2010b; Xu et al., 2010; Chen and Li, 2011; Liu, 2016) used Sprague Dawley (SD) rats; 1 study (Song et al., 2013) used Wistar rats; 8 studies (Choi et al., 2011; Hwang et al., 2014; Wang et al., 2014; Oh et al., 2015; Bao et al., 2016; Ma et al., 2017; Zheng et al., 2017; Delgado et al., 2019) used Institute of Cancer Research (ICR) mice; 6 studies (Li et al., 2009; Xu et al., 2011; Zhao et al., 2014; Qi et al., 2014; Wang et al., 2015; Shi et al., 2016) used Kunming (KM) mice; 3 studies (Gao et al., 2011; Liu et al., 2015; Yao, 2016) used unknown breed mice. Twenty-seven studies (Feng et al., 2009a; Feng et al., 2009b; Feng et al., 2009c; Li et al., 2009; Pan et al., 2009; Feng et al., 2010a; Feng et al., 2010b; Feng et al., 2010c; Pan et al., 2010a; Pan et al., 2010b; Xu et al., 2010; Chen and Li, 2011; Gao et al., 2011; Choi et al., 2011; Song et al., 2013; Hwang et al., 2014; Wang et al., 2014; Liu et al., 2015; Wang et al., 2015; Oh et al., 2015; Bao et al., 2016; Liu, 2016; Shi et al., 2016; Yao, 2016; Ma et al., 2017; Zheng et al., 2017; Delgado et al., 2019) used male animals, 2 studies (Qi et al., 2014; Zhao et al., 2014) used both female and male rats, and 1 study (Xu et al., 2011) did not mention gender of animals. The weight of adult rats varied between 160 and 260 g and mice between 16 and 28 g. Anesthetic was mentioned in 1 study (Chen and Li, 2011). Ginsenosides were used in 18 studies (Feng et al., 2009a; Feng et al., 2009b; Feng et al., 2009c; Li et al., 2009; Pan et al., 2009; Feng et al., 2010a; Feng et al., 2010b; Feng et al., 2010c; Pan et al., 2010a; Pan et al., 2010b; Xu et al., 2010; Chen and Li, 2011; Song et al., 2013; Liu et al., 2015; Oh et al., 2015; Wang et al., 2015; Liu, 2016; Yao, 2016), ginseng in 6 studies (Choi et al., 2011; Gao et al., 2011; Hwang et al., 2014; Zhao et al., 2014; Shi et al., 2016; Ma et al., 2017), ginseng oligopeptides in 2 studies (Bao et al., 2016; Delgado et al., 2019), ginseng polysaccharides in 2 studies (Wang et al., 2014; Zheng et al., 2017) and ginseng protein in 2 studies (Xu et al., 2011; Qi et al., 2014). Thirteen fatigue models (Feng et al., 2009a; Feng et al., 2009b; Feng et al., 2009c; Pan et al., 2009; Feng et al., 2010a; Feng et al., 2010b; Feng et al., 2010c; Pan et al., 2010a; Pan et al., 2010b; Xu et al., 2010; Chen and Li, 2011; Hwang et al., 2014; Liu, 2016) were produced by Horizontal treadmill exercise, 10 models (Li et al., 2009; Gao et al., 2011; Xu et al., 2011; Zhao et al., 2014; Liu et al., 2015; Oh et al., 2015; Wang et al., 2015; Shi et al., 2016; Yao, 2016; Ma et al., 2017) by weight-loaded swimming test (WLST), 6 models (Song et al., 2013; Qi et al., 2014; Wang et al., 2014; Bao et al., 2016; Zheng et al., 2017; Delgado et al., 2019) by Forced swimming test (FST) and 3models (Choi et al., 2011; Oh et al., 2015; Shi et al., 2016) by Rota-rod test. The non-functional liquid, normal saline or no treatment control was introduced in all 30 studies. Thirteen studies (Li et al., 2009 Xu et al., 2011; Song et al., 2013; Qi et al., 2014; Zhao et al., 2014; Liu et al., 2015; Oh et al., 2015; Wang et al., 2015; Bao et al., 2016; Shi et al., 2016; Yao, 2016; Ma et al., 2017; Delgado et al., 2019) utilized swimming time as outcome measure, and climbing time in 1 study (Shi et al., 2016). Serum biochemical parameters, including BLA, BUN, glucose (GLU), creatine kinase (CK), MDA, superoxide dismutase (SOD), glutathione peroxidase (GSH-Px), and LDH were reported in 21 studies (Feng et al., 2009c; Li et al., 2009; Pan et al., 2010a; Choi et al., 2011; Gao et al., 2011; Xu et al., 2011; Song et al., 2013; Hwang et al., 2014; Qi et al., 2014; Wang et al., 2014; Zhao et al., 2014; Liu et al., 2015; Oh et al., 2015; Wang et al., 2015; Bao et al., 2016; Liu, 2016; Shi et al., 2016; Yao, 2016; Ma et al., 2017; Zheng et al., 2017; Delgado et al., 2019); skeletal muscle biochemical parameters, including glycogen, MDA, SOD, catalase (CAT), and GSH-Px in 13 studies (Li et al., 2009; Feng et al., 2010a; Feng et al., 2010b; Feng et al., 2010c; Pan et al., 2010b; Xu et al., 2010; Gao et al., 2011; Zhao et al., 2014; Wang et al., 2015; Bao et al., 2016; Liu, 2016; Ma et al., 2017; Delgado et al., 2019); liver biochemical parameters, including glycogen, MDA, SOD, and GSH-Px in 14 studies (Feng et al., 2009c; Pan et al., 2010a; Gao et al., 2011; Xu et al., 2011; Hwang et al., 2014; Qi et al., 2014; Zhao et al., 2014; Liu et al., 2015; Wang et al., 2015; Bao et al., 2016; Shi et al., 2016; Yao, 2016; Ma et al., 2017; Delgado et al., 2019); brain tissue biochemical parameters, including acetylcholine (Ach), dopamine (DA), gamma-aminobutyric acid (GABA), 5-hydroxytryptamine (5-HT) in 5 study (Feng et al., 2009a; Feng et al., 2009b; Feng et al., 2009c; Chen and Li, 2011; Liu, 2016); and body weight in 7 studies (Li et al., 2009; Xu et al., 2011; Song et al., 2013; Hwang et al., 2014; Bao et al., 2016; Liu, 2016; Shi et al., 2016). Approach to achieving of PGC was reported in 15 studies (Feng et al., 2009a; Feng et al., 2009b; Feng et al., 2009c; Li et al., 2009; Pan et al., 2009; Feng et al., 2010a; Feng et al., 2010b; Feng et al., 2010c; Pan et al., 2010a; Pan et al., 2010b; Xu et al., 2010; Chen and Li, 2011; Choi et al., 2011; Song et al., 2013; Hwang et al., 2014; Wang et al., 2014; Liu et al., 2015; Oh et al., 2015; Wang et al., 2015; Bao et al., 2016; Liu, 2016; Shi et al., 2016; Zheng et al., 2017; Delgado et al., 2019) and quality control of PGC was reported in 8 studies (Feng et al., 2009c; Feng et al., 2010c; Chen and Li, 2011; Wang et al., 2014; Liu et al., 2015; Oh et al., 2015; Zheng et al., 2017; Delgado et al., 2019). **Tables 3** and **4** show the characteristics of included publications.

**Table 3:** Characteristics of the included animal studies.

Study (years)	Species Sex Weight N	Anesthetic	Model (method)	Experimental group (drugs, concentration, administration, duration)	Control group	Outcome measure	Intergroup differences*
Xu et al., 2011	KM mice NG 18–22 g 240	–	weight-loaded swimming (WLS) test	ginseng protein (0.1, 0.2, 0.4 g/kg) ig, 30 days	Distilled water for same volume	Mice body weight Swimming time in mice BLA Liver glycogen Serum urea nitrogen	P>0.05 P>0.05 P<0.05 P>0.05 P>0.05
Wang et al., 2010	ICR mice M (11–12 weeks) 104	–	Forced swimming test	WGP (50,100,200 mg/kg) WGPA(40,100,160,200 mg/kg) WGPN(40,100,160,200 mg/kg) ig, 15 days	NS for same volume	Serum GLU Serum TG Serum CK Serum LDH Serum MDA Serum SOD Serum GSH-Px	P<0.01 P<0.01 P<0.05 P<0.05 P<0.001 P<0.05 P<0.05
Wang et al., 2013	ICR mice M (11–12 weeks) 104	–	Forced swimming test	WGPA (200 mg/kg) WGPA-A(200 mg/kg) WGPA-N(200 mg/kg) ig, 15 days	NS for same volume	Serum MDA Serum SOD SerumGSH-Px Serum LDH	P<0.001 P<0.01 P<0.01 P<0.05
Song et al., 2013	Wistar rat M 180–220 g 60	–	Forced swimming test	PTS (25, 50, 100 mg/kg) ig, 7 weeks	NS for same volume	Mice body weight Swimming time in mice Serum GLU Serum urea nitrogen Blood lactic acid	P>0.05 P<0.05 P<0.05 P<0.05 P<0.05
Xu et al., 2010	SD rat M 180–200 g 30	–	Horizontal treadmill exercise	Ginsenoside Rb1(50 mg/kg) ig, 14 days	NS for same volume	Skeletal muscle MDA Skeletal muscle SOD	P<0.01 P<0.05
Feng et al., 2010a	SD rat M 180–220 g 40	–	Horizontal treadmill exercise	Ginsenoside (50 mg/kg) Ginsenoside Rb1(50 mg/kg) ig, 14 days	NS for same volume	Calcium content in skeletal muscle cell Mitochondrial membrane potential Skeletal muscle MDA Skeletal muscle SOD Skeletal muscle GLU	P<0.05 P<0.05 P<0.05 P<0.05 P<0.05
Feng et al., 2009a	SD rat M 180–220 g 40	–	Horizontal treadmill exercise	Ginsenoside (50 mg/kg) Ginsenoside Re (50 mg/kg) ig, 14 days	NS for same volume	Hypothalamus Ach Hypothalamus DA Hypothalamus 5-HT Hypothalamus GABA	P<0.01 P<0.01 or P<0.05 P<0.01 P<0.01
Liu, 2016	SD rat M 240 ± 20 g 40	–	Horizontal treadmill exercise	Ginsenoside Rg1(30 mg/kg) ig, 8 weeks	NS for same volume	Mice body weight Exhaustion time Skeletal muscle SOD Skeletal muscle GSH-Px Skeletal muscle CAT Skeletal muscle MDA Skeletal muscle carbonylation protein content Brain tissue SOD Brain tissue GSH-Px Brain tissue CAT Brain tissue MDA Brain tissue carbonylation protein content Blood lactic acid	P>0.05 P<0.01 P<0.01 P<0.05 P<0.05 P<0.05 P<0.05 P<0.01 P<0.05 P<0.05 P>0.05 P<0.05 13. P>0.05
Feng et al., 2010b	SD rat M 180–220 g 40	–	Horizontal treadmill exercise	Ginsenoside Rb1(50 mg/kg) Ginsenoside Rg1(50 mg/kg) ig, 14 days	NS for same volume	Hypothalamus Ach Hypothalamus DA Hypothalamus 5-HT Hypothalamus GABA	P<0.01 P<0.01 P<0.01 P<0.01
Wang et al., 2015	KM mice M 20 ± 2 g 180	–	weight-loaded swimming (WLS) test	Ginsenoside Rg1(5, 10, 20, 40, 80 mg/kg) ig, 2 weeks	NS for same volume	Swimming time Blood lactic acid Serum BUN Serum GLU Muscle glycogen Hepatic glycogen	P>0.05 P>0.05 P>0.05 P>0.05 P<0.01 P<0.05
Yao, 2016	mice M 19–22 g 80	–	weight-loaded swimming (WLS) test	Ginsenoside (0.8, 1.6, 3.2 g/kg) ig, 28 days	distilled water for same volume	Serum BUN Serum LDH Serum BLA Serum CK Hepatic glycogen Liver MDA Liver SOD Liver GSH-Px Swimming time	P<0.05 P<0.05 P<0.05 P<0.05 P<0.05 P<0.05 P<0.05 P<0.05 P<0.05
Liu et al., 2015	mice M 18–22 g 40	–	weight-loaded swimming (WLS) test	Ginsenoside (0.8, 1.6, 3.2 g/kg) ig, 28 days	distilled water for same volume	Serum BUN Serum LDH Serum BLA Serum CK Hepatic glycogen Liver MDA Liver SOD Liver GSH-Px Swimming time	P<0.001 P<0.01 P<0.05 P<0.001 P<0.01 P<0.01 P>0.05 P>0.05 P<0.01
Feng et al., 2009b	SD rat M 180–220 g 30	–	Horizontal treadmill exercise	Ginsenoside Re(50 mg/kg) ig, 14 days	NS for same volume	1.Serum MDA Liver MDA Skeletal muscle MDA Serum SOD Liver SOD Skeletal muscle SOD	P<0.01 P<0.01 P<0.01 P<0.01 P<0.01 P<0.01
Feng et al., 2010c	SD rat M 180–220 g 30	–	Horizontal treadmill exercise	Ginsenoside Rg1(50 mg/kg) ig, 14 days	NS for same volume	Calcium content in skeletal muscle cell Mitochondrial membrane potential Skeletal muscle SOD Skeletal muscle MDA	P<0.01 P<0.01 P<0.01 P<0.01
Pan et al., 2010a	SD rat M 180–220 g 30	–	Horizontal treadmill exercise	Ginsenosides (50 mg/kg) ig, 14 days	NS for same volume	Calcium content in skeletal muscle cell Mitochondrial membrane potential Skeletal muscle SOD Skeletal muscle MDA	P<0.01 P<0.01 P<0.01 P<0.01
Chen and Li, 2011	SD rat M 160–200 g 45	Celiac anesthesia with chloral hydrate	Horizontal treadmill exercise	Ginsenosides (70 mg/kg) ig, 8 weeks	NS for same volume	Hippocampus tissue GABA Hippocampus tissue Ach Hippocampus tissue NE Hippocampus tissue DA Hippocampus tissue 5-HT DA/5-HT	P<0.05 P<0.05 P<0.05 P<0.05 P<0.05 P<0.05
Pan et al., 2009	SD rat M 180–220 g 40	–	Horizontal treadmill exercise	Ginsenosides(50 mg/kg) Ginsenoside Rg1(50 mg/kg) ig, 14 days	NS for same volume	Hippocampus tissue GABA Hippocampus tissue DA Hippocampus tissue 5-HT Hippocampus tissue Ach	P<0.01 P<0.05 P<0.01 P<0.05
Li et al., 2009	KM mice M 18–22 g 80	–	weight-loaded swimming (WLS) test	Pseudo- ginsenoside GQ(PGQ) (0.2, 0.4, 0.8 g/kg) ig, 5 weeks	NS for same volume	Mice body weight Swimming time in mice Blood lactic acid Serum BUN Serum LDH Muscle glycogen	P<0.05 P<0.01 P<0.05 P<0.05 P<0.05 P<0.05
Feng et al., 2009c	SD rat M 180–220 g 40	–	Horizontal treadmill exercise	Ginsenoside Rb1(50 mg/kg) Ginsenoside Re(50 mg/kg) ig, 14 days	NS for same volume	Hippocampus tissue DA Hippocampus tissue 5-HT Hippocampus tissue GABA Hippocampus tissue Ach	1.P<0.05 P<0.05 P<0.01 P<0.01
Pan et al., 2010b	SD rat M 180–220 g 30	–	Horizontal treadmill exercise	Ginsenoside Rb1 (50 mg/kg) ig, 14 days	NS for same volume	Serum MDA Serum SOD Liver MDA Liver SOD	P<0.01 P<0.01 P<0.01 4. P<0.01
Bao et al., 2016	ICR mice M 18–22 g 240	–	Forced Swimming Test	GOP (125, 250, 500 mg/kg) ig, 30 days	Distilled water for same volume	Mice body weight Swimming time in mice Serum LDH Serum Urea Nitrogen Hepatic Glycogen Blood Lactic Acid skeletal muscle SOD skeletal muscles CAT skeletal muscles MDA NRF-1 TFAM mtDNA	P>0.05 P<0.01 P<0.01 P<0.01 P<0.01 P<0.01 P<0.01 P<0.01 P<0.01 P<0.05 P<0.05 P<0.05
Oh et al., 2015	ICR mice M 26−28 g 49	–	weight-loaded swimming (WLS) test and Rota-rod test	PPD (5, 10 mg/kg) PPT (5, 10 mg/kg) ig, 5 days	Distilled water for same volume	Swimming time Serum corticosterone Serum GLU Serum lactate Serum LDH Serum FFA Serum creatinine Serum corticosterone Serum lactate Serum FFA Serum creatinine	P<0.05 or P<0.01 P<0.05 or P<0.01 P<0.05 or P<0.01 P<0.05 or P<0.01 P<0.05 or P<0.01 P<0.05 or P<0.01 P<0.05 or P<0.01 P<0.01 P<0.05 P<0.05 P<0.05
Qi et al., 2014	KM mice F/M 20 ± 2 g 80	–	Forced swimming test	AGP (125, 250, 500 mg/kg) ig, 28 days	Distilled water for same volume	Swimming time BLA SUN hepatic glycogen Serum GSH-Px Serum SOD Serum MDA	P<0.01 P<0.01 P<0.01 P<0.01 P<0.01 P<0.01 P<0.01
Li et al., 2018	ICR mice M 18–22 g 240	–	Forced swimming test	QOPs (225, 450, 900 mg/kg) ig, 30 days	NS for same volume	Swimming time Serum LDH BUN Hepatic glycogen BLA skeletal muscle SOD skeletal muscles GSH-Px skeletal muscles MDA Na+-K+-ATPase Ca2+-Mg2+- ATPase SDH NRF-1 TFAM mtDNA	P<0.01 P<0.01 P<0.01 P<0.01 P<0.01 P<0.01 P<0.01 P<0.01 P<0.01 P<0.01 P<0.01 P<0.01 P<0.01 P<0.01
Zhao et al., 2014	KM mice F/M 18–22 g 70	–	weight-loaded swimming (WLS) test	Ginseng (0.2 ml/10 g) Red Ginseng (0.2 ml/10 g) mild property ginseng (0.2 ml/10 g) ig, 14 days	Distilled water for same volume	Swimming time hypoxia tolerance time BLA Hepatic glycogen muscle glycogen Serum BUN	P<0.01 P<0.01 P<0.01 P<0.01 P<0.01 P<0.01
Gao et al., 2011	mice M 18–22 g 120	–	weight-loaded swimming (WLS) test	Ginseng (1.25, 2.5, 5 mg/kg) Red Ginseng (1.25, 2.5, 5 mg/kg)	NS for same volume	BLA Serum BUN Serum SOD Serum LDH Hepatic glycogen muscle glycogen	P<0.05 P<0.05 P>0.05 P>0.05 P<0.01 P<0.01
Ma et al., 2017	ICR mice M NG 24	–	Forelimb Grip Strength/weight-loaded swimming (WLS) test	CMG extract (5, 25 mg/kg) ig, 4 weeks	Distilled water for same volume	Forelimb grip strength Swimming time Serum Lactate Serum NH3 Serum CK Serum GLU Serum BUN Hepatic glycogen muscle glycogen Serum LDH organ index	P<0.01 P<0.01 P<0.01 P<0.01 P<0.01 P<0.01 P<0.01 P<0.01 P>0.05 P<0.01 P>0.05 P>0.05
Choi et al., 2011	ICR mice M 17–20 g 40	–	restraint stress/Overcoming electroshock/Cold swimming test/Rota-rod test	RG (50, 100, 200, 400 mg/kg) ig, 7 days	NR	Serum CORT Blood lactic acid	P<0.05 P>0.05
Hwang et al., 2014	ICR mice M NG 43	–	aerobic running exercise	RG(1 g/kg) ig, 2 weeks	Distilled water for same volume	1.body weight Carbohydrate Oxidation Fat Oxidation Serum Free fatty acid Serum Glucose SerumInsulin Liverglycogen Gastrocnemius-white muscle glycogen Gastrocnemius-red muscle glycogen	P>0.05 P>0.05 P>0.05 P<0.05 P>0.05 P>0.05 P<0.05 P>0.05 P>0.05
Shi et al., 2016	KM mice M 18–22 g 20	–	weight-loaded swimming (WLS) test and Rota-rod test	BG(1.65, 3.30, 4.95 g/kg) ig, 2 weeks	Distilled water for same volume	body weight pole-climbing time swimming time liverglycogen blood lactic acid Serum LDH	P>0.05 P<0.001 P<0.001 P<0.01 P<0.01 P<0.01

5-HT, 5-hydroxytryptamine; Ach, acetylcholine; BLA, Blood lactic acid; BUN, blood urea nitrogen; CK, creatine phosphokinase; CORT, corticosterone; DA, dopamine; GABA, gamma-aminobutyric acid; GLU, glucose; GSH-Px, glutathione peroxidase; LDH, lactic dehydrogenase; MDA, malondialdehyde; mtDNA, mitochondrial DNA; NRF-1, Nuclear respiratory factor 1; PTS, panaxtrol saponin; SDH, succinate dehydrogenase; SOD, superoxide dismutase; TG, triglyceride; TFAM, Mitochondrial transcription factor A; WGP, water-soluble ginseng polysaccharides; WGPA, ginseng pectin; WGPN, starch-like glucans.

**Table 4:** Statement of the characteristics of *Panax ginseng* C. A. Mey and its compounds(b).

Study	Herb source	Type of herbal or bioactive compound	Purity	Approach to achieving	Quality control (lot number)	Chemical analysis	Structure or Composition
Xu et al., 2011	*Panax ginseng* C.A. Mey	Ginseng proteins	80%	not reported	Not reported	included-UC	–
Wang et al., 2010	*Panax ginseng* C.A. Mey	Ginseng polysaccharides	not reported	the School of Life Sciences, Northeast Normal University, China	reported (20081001)	included-HPLC	–
Wang et al., 2013	*Panax ginseng* C.A. Mey	Ginseng pectin polysaccharide	not reported	the School of Life Sciences, Northeast Normal University, China	reported (20081001)	included-HPLC	–
Song et al., 2013	*Panax ginseng* C.A. Mey	PTS	not reported	Department of Pathophysiology, Basic Medical College of Jilin University, Jilin, China	not reported	not included	–
Xu et al., 2010	*Panax ginseng* C.A. Mey	Ginsenoside Rb1	not reported	Shanghai Tongtian Biotechnology Co., Ltd., Shanghai, China	not reported	included-HPLC	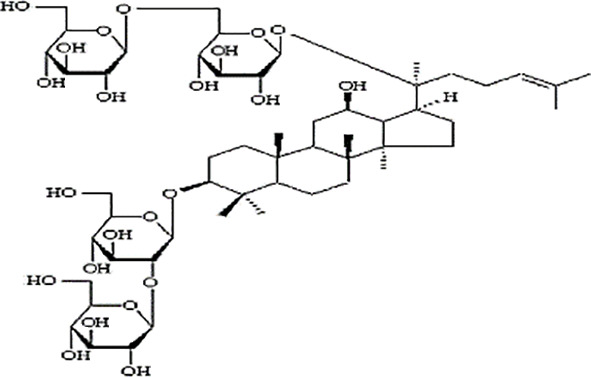
Feng et al., 2010a	*Panax ginseng* C.A. Mey	Ginsenoside Ginsenoside Rb1	not reported	Shanghai Tongtian Biotechnology Co., Ltd., Shanghai, China	not reported	included-HPLC	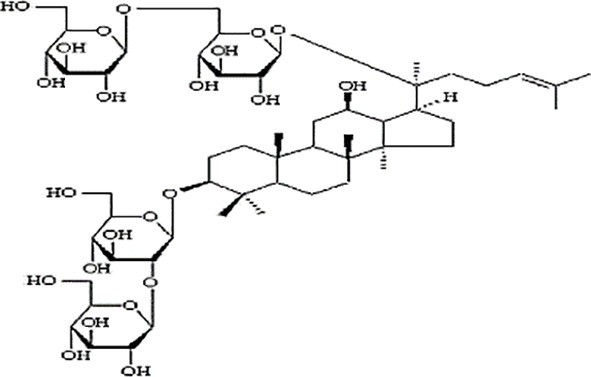
Feng et al., 2010a	*Panax ginseng* C.A. Mey	Ginsenoside Ginsenoside Re	not reported	Shanghai Tongtian Biotechnology Co., Ltd., Shanghai, China	not reported	included-HPLC	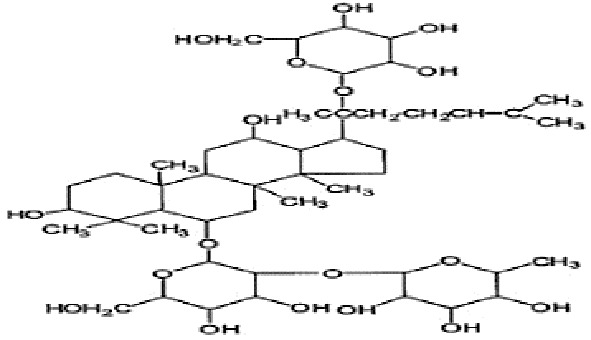
Liu, 2016	*Panax ginseng C.A. Mey*	Ginsenoside Rg1	98.59%	Dingguo Biotechnology Co., Ltd., China	not reported	included-HPLC	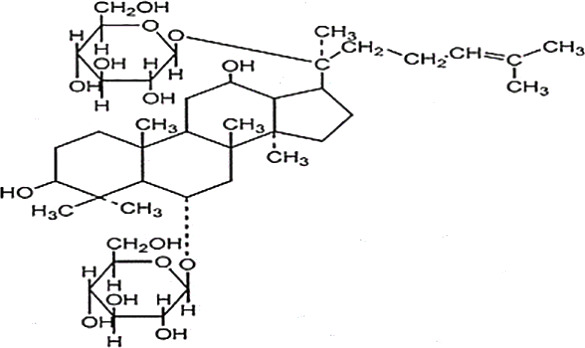
Feng et al., 2010b	*Panax ginseng* C.A. Mey	Ginsenoside Rb1 Ginsenoside Rg1	not reported	Shanghai Tongtian Biotechnology Co., Ltd., Shanghai, China	not reported	included-HPLC	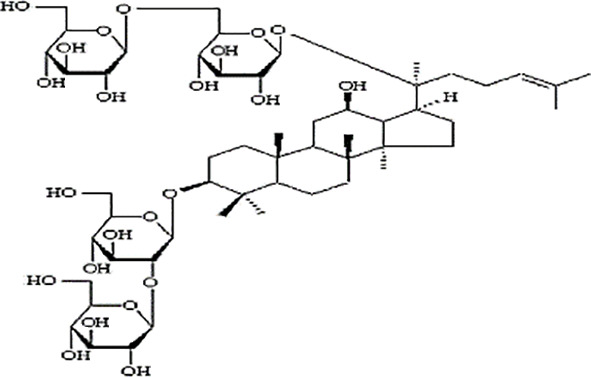 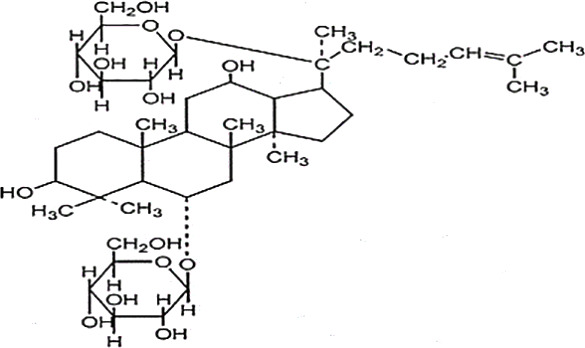
Wang et al., 2015	*Panax ginseng* C.A. Mey	Ginsenoside Rg1	98%	Department of Organic Chemistry, Basic Medical College of Jilin University, Jilin, China	not reported	included-HPLC	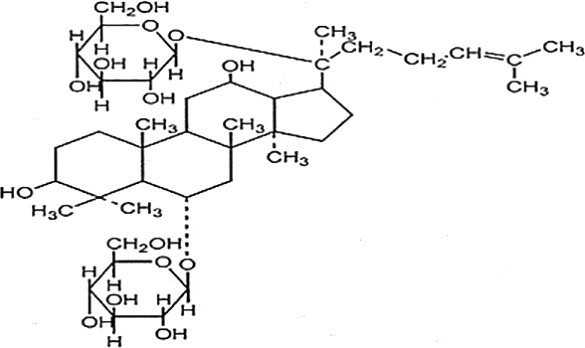
Yao, 2016	*Panax ginseng* C.A. Mey	ginsenoside	not reported	not reported	not reported	not included	
Liu et al., 2015	*Panax ginseng* C.A. Mey	Ginsenoside	> 80%	Xi'an Ruiying Biotechnology Co., Ltd., Xi'an, China	reported (RYRS20140325)	not included	
Feng et al., 2009b	*Panax ginseng* C.A. Mey	Ginsenoside Re	not reported	Shanghai Tongtian Biotechnology Co., Ltd., Shanghai, China	Reported-according to (07120623)	included-HPLC	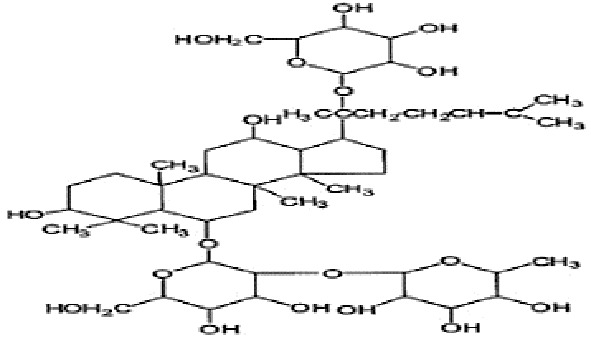
Feng et al., 2010c	*Panax ginseng* C.A. Mey	Ginsenoside Rg1	not reported	Shanghai Tongtian Biotechnology Co., Ltd., Shanghai, China	reported (07120624)	included-HPLC	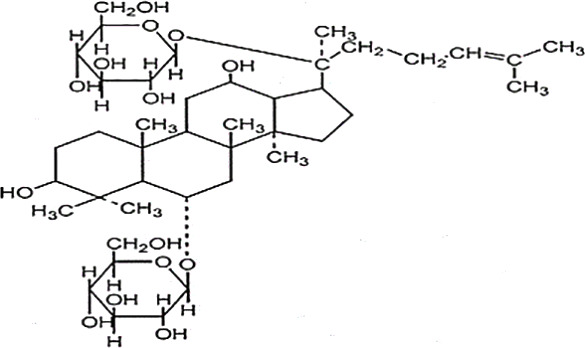
Pan et al., 2010a	*Panax ginseng* C.A. Mey	Ginsenosides	not reported	Shanghai Tongtian Biotechnology Co., Ltd., Shanghai, China	not reported	included-HPLC	–
Chen and Li, 2011	*Panax ginseng* C.A. Mey	Ginsenosides	not reported	Jilin Yawei Pharmaceutical Co., Ltd., Jilin, China	Reported (94032019)	included-HPLC	–
Pan et al., 2009	*Panax ginseng* C.A. Mey	Ginsenosides Ginsenoside Rg1	not reported	Shanghai Tongtian Biotechnology Co., Ltd., Shanghai, China	not reported	included-HPLC	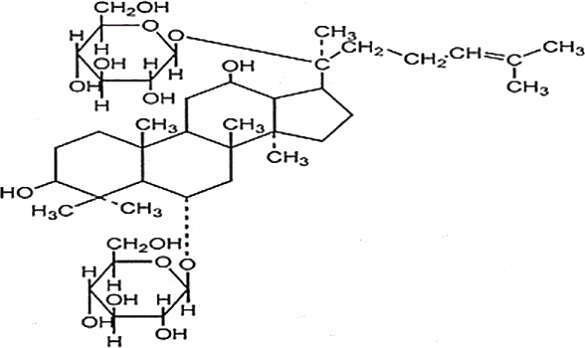
Li et al., 2009	*Panax ginseng* C.A. Mey	Pseudo- ginsenoside GQ Rg	98.7%	Institute of Regenerative Medicine, Jilin University, Jilin, China	not reported	not included	–
Feng et al., 2010c	*Panax ginseng* C.A. Mey	Ginsenoside Rb1 Ginsenoside Re	not reported	Shanghai Tongtian Biotechnology Co., Ltd., Shanghai, China	not reported	included-HPLC	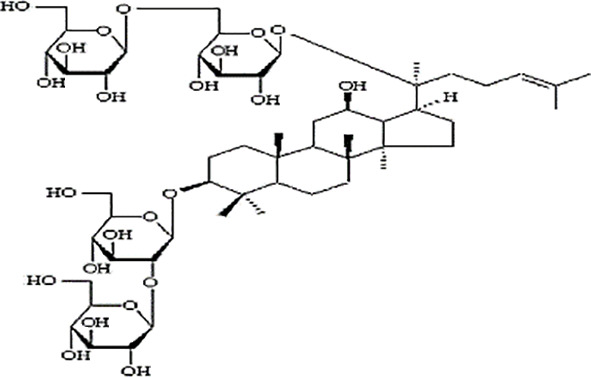 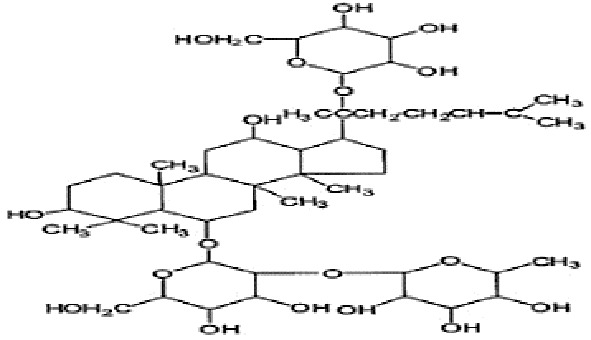
Pan et al., 2010b	*Panax ginseng* C.A. Mey	Ginsenoside Rb1	not reported	Shanghai Tongtian Biotechnology Co., Ltd., Shanghai, China	not reported	included-HPLC	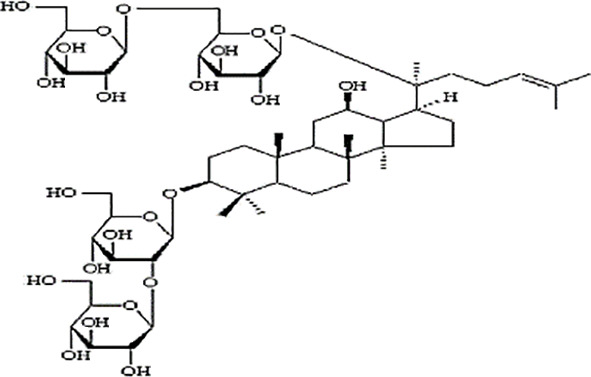
Bao et al., 2016	*Panax ginseng* C.A. Mey	ginseng oligopeptides (GOP)	95.42%	Jilin Taigu Biological Engineering Co., Ltd., Jilin, China	not reported	included-HPLC	–
Oh et al., 2015	*Panax ginseng* C.A. Mey	20(S)-Protopanaxadiol(PPD) 20(S)-Protopanaxatriol(PPT)	≥98%	the College of Pharmacy, Kyung HeeUniversity, Geumsan, Korea	reported (KHUP201409201)	Included-HPLC	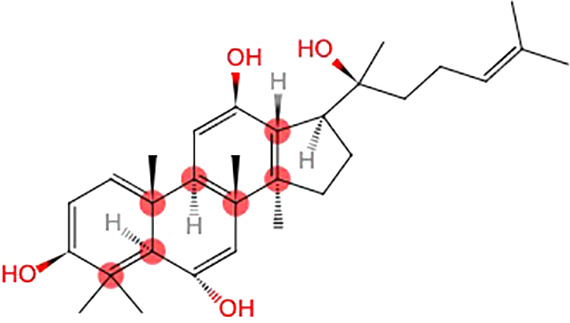 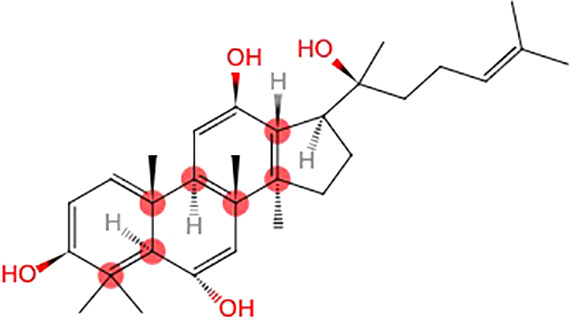
Qi et al., 2014	Panaxquinquefolium	American ginseng proteins (AGP)	80%	Agilent Technologies, USA	not reported	included-HPLC	–
Li et al., 2018	Panaxquinquefolium	small-molecule oligopeptides isolated from American ginseng(QOPs)	96.46%	SinoMed Peptide Valley Bioengineering Co., Ltd., China	reported (CN105154509A)	included-HPLC	–
Zhao et al., 2014	Red ginseng	Ginseng Red Ginseng mild property ginseng	not reported	not reported	not reported	not included	Red GinsengContain 4.015% ginsenosides (Rg1 0.262%, Re 0.145%, Rf 0.151%, Rh1 0.284%, Rg2 0.402%, Rb1 0.394%, Rc 0.318%, Rb2 0.247%, Rb3 0.465%, Rd 0.437%, Rg3 0.412%, Rk1 0.194%, Rg5 0.304%) HPLC data from Kim et al., 2007
Gao et al., 2011	*Panax ginseng* C.A. Mey	Ginseng Red Ginseng	not reported	not reported	not reported	not included	Red GinsengContain 4.015% ginsenosides (Rg1 0.262%, Re 0.145%, Rf 0.151%, Rh1 0.284%, Rg2 0.402%, Rb1 0.394%, Rc 0.318%, Rb2 0.247%, Rb3 0.465%, Rd 0.437%, Rg3 0.412%, Rk1 0.194%, Rg5 0.304%) HPLC data from Kim et al., 2007
Ma et al., 2017	*Panax ginseng* C.A. Mey	Changbai Mountain Ginseng(CMG)	not reported	not reported	not reported	included-HPLC	Contain 11.5% ginsenosides (Ra1 0.46%, Ra2 0.69%, Rb1 0.22%, Rb2 0.94%, Rb3 1.22%, Rc 0.33%, Rd 1.54%, Re 0.11%, Rf 1.14%, Rg1 0.2%, Ro 1.19%, 20-glc-G-Rf 1.60%, R1 0.71%, R2 1.15%) HPLC data from Wang et al. 2017
Choi et al., 2011	Red ginseng	Ren Ginseng	not reported	Korea Ginseng Corp., Seoul, Korea	not reported	included-HPLC	Contain 4.015% ginsenosides (Rg1 0.262%, Re 0.145%, Rf 0.151%, Rh1 0.284%, Rg2 0.402%, Rb1 0.394%, Rc 0.318%, Rb2 0.247%, Rb3 0.465%, Rd 0.437%, Rg3 0.412%, Rk1 0.194%, Rg5 0.304%) HPLC data from Kim et al., 2007
Hwang et al., 2014	Red ginseng	Ren Ginseng	not reported	Korea Ginseng Corp., Seoul, Korea	not reported	included-HPLC	Contain 1.964% ginsenosides(Rg1 0.071%, Re 0.093%, Rf 0.121%, Rf1 0.078%, Rg2(s) 0.192%, Rg2 0.129%, Rb1 0.462%, Rc 0.241%, Rb2 0.183%, Rd 0.089%, Rg3(s) 0.214%, Rg3 0.091%) HPLC data from Hwang et al., 2014
Shi et al., 2016	Black ginseng	black Ginseng	not reported	HunchunXuzhu Trading Co., Ltd., Jilin, China	not reported	not included	Contain 3.341% ginsenosides (Rg1 0.232%, Re 0.186%, Rf 0.158%, Rb1 0.184%, Rc 0.145%, Rb2 0.174%, Rd 0.169%, Rg6 0.138%, F4 0.127%, Rk3 0.162%, Rh4 0.102%, 20(S)-Rg3 0.173%, 20(R)-Rg3 0.121%, 20(S)-Rs3 0.146%, 20(R)-Rs3 0.213%, Rk1 0.178%, Rg5 0.321%, Rs5 0.225%, Rs4 0.187%) HPLC data from Sun et al., 2009

HPLC, high-performance liquid chromatography; PGC, ginseng and its compounds.

### Study Quality

#### Randomized Clinical Trials

**Table 5** illustrates the methodological quality of 8 RCTs based on the Cochrane Collaboration's tool. All of them were ranged from 4 to 7 points. All included studies reported the method of random sequences generation, the criteria of a double-blind study design, and taking the complete outcome data into account. Three studies (Hartz et al., 2004; Hyeong-Geug et al., 2013; Lee et al., 2016) reported using allocation concealment. Two studies (Hartz et al., 2004; Lee et al., 2016) applied blinding specifically during outcome measure assessment. The protocols of 3 studies (Hyeong-Geug et al., 2013; Kim et al., 2016; Lee et al., 2016) were registered in the Clinical Trial Registry. In other bias, all eight studies were supported by nonprofit institutions and accounted for baseline comparability, but no study provided sample size estimation information.

**Table 5:** The methodological quality of included randomized control trials.

included studies	A	B	C	D	E	F	G	Total score
Engels et al., 1996	+	?	+	?	+	?	+	4+
Engels et al., 2001	+	?	+	?	+	?	+	4+
Engels et al., 2003	+	?	+	?	+	?	+	4+
Hartz et al., 2004	+	+	+	+	+	?	+	6+
Hyeong-Geug et al., 2013	+	+	+	?	+	+	+	6+
Kim et al., 2016	+	?	+	?	+	+	+	5+
Lee et al., 2016	+	+	+	+	+	+	+	7+
Gal et al., 1996	+	?	+	?	+	?	+	4+

A, random sequence generation; B, allocation concealment; C, blinding of participants and personnel; D, blinding of outcome assessment; E, incomplete outcome data; F, selective reporting; G, other sources of bias.

#### Animal Studies

The quality scores of studies included varied from 4 to 7 out of 10 points with the average of 5.73. Of which, 4 studies (Feng et al., 2009b; Wang et al., 2015; Liu, 2016; Yao, 2016) got 4 points; 6 studies (Li et al., 2009; Xu et al., 2010; Chen and Li, 2011; Xu et al., 2011; Liu et al., 2015; Shi et al., 2016) got 5 points; 13 studies (Feng et al., 2009a; Feng et al., 2009b; Feng et al., 2010a; Feng et al., 2010b; Feng et al., 2010c; Pan et al., 2010a; Pan et al., 2010b; Choi et al., 2011; Gao et al., 2011; Song et al., 2013; Wang et al., 2014; Zhao et al., 2014; Zheng et al., 2017) got 6 points; and 6 studies (Hwang et al., 2014; Qi et al., 2014; Oh et al., 2015; Bao et al., 2016; Ma et al., 2017; Delgado et al., 2019) got 7 points. All the included records described appropriate animal models (aged or female involved) and used an anesthetic without significant intrinsic neuroprotective activity. However, no study reported blinded induction of model, blinding their assessment of outcome, and a sample size calculation. Twenty-nine studies (Feng et al., 2009a; Feng et al., 2009b; Feng et al., 2009c; Li et al., 2009; Pan et al., 2009; Feng et al., 2010a; Feng et al., 2010b; Feng et al., 2010c; Pan et al., 2010a; Pan et al., 2010b; Xu et al., 2010; Chen and Li, 2011; Choi et al., 2011; Gao et al., 2011; Xu et al., 2011; Song et al., 2013; Hwang et al., 2014; Qi et al., 2014; Wang et al., 2014; Zhao et al., 2014; Liu et al., 2015; Oh et al., 2015; Wang et al., 2015; Bao et al., 2016; Shi et al., 2016; Yao, 2016; Ma et al., 2017; Zheng et al., 2017; Delgado et al., 2019) were peer-reviewed publications. Twenty-seven studies (Feng et al., 2009a; Feng et al., 2009b; Feng et al., 2009c; Li et al., 2009; Pan et al., 2009; Feng et al., 2010a; Feng et al., 2010b; Feng et al., 2010c; Pan et al., 2010a; Pan et al., 2010b; Xu et al., 2010; Chen and Li, 2011; Gao et al., 2011; Xu et al., 2011; Song et al., 2013; Hwang et al., 2014; Qi et al., 2014; Zhao et al., 2014; Liu et al., 2015; Oh et al., 2015; Wang et al., 2015; Bao et al., 2016; Liu, 2016; Shi et al., 2016; Yao, 2016; Ma et al., 2017; Delgado et al., 2019) allocated randomly to treatment group and control group. Twenty-four studies (Feng et al., 2009a; Feng et al., 2009b; Pan et al., 2009; Feng et al., 2010a; Feng et al., 2010b; Feng et al., 2010c; Pan et al., 2010a; Pan et al., 2010b; Xu et al., 2010; Chen and Li, 2011; Gao et al., 2011; Choi et al., 2011; Song et al., 2013; Hwang et al., 2014; Qi et al., 2014; Wang et al., 2014; Zhao et al., 2014; Oh et al., 2015; Bao et al., 2016; Liu, 2016; Shi et al., 2016; Ma et al., 2017; Zheng et al., 2017; Delgado et al., 2019) described control of temperature. Twenty-four studies (Feng et al., 2009a; Feng et al., 2009b; Li et al., 2009; Pan et al., 2009; Feng et al., 2010a; Feng et al., 2010b; Feng et al., 2010c; Pan et al., 2010a; Pan et al., 2010b; Choi et al., 2011; Gao et al., 2011; Xu et al., 2011; Song et al., 2013; Hwang et al., 2014; Qi et al., 2014; Wang et al., 2014; Zhao et al., 2014; Liu et al., 2015; Oh et al., 2015; Bao et al., 2016; Liu, 2016; Ma et al., 2017; Zheng et al., 2017; Delgado et al., 2019) declared no potential conflict of interests, and 9 studies (Choi et al., 2011; Hwang et al., 2014; Qi et al., 2014; Wang et al., 2014; Oh et al., 2015; Bao et al., 2016; Ma et al., 2017; Zheng et al., 2017; Delgado et al., 2019) reported compliance with animal welfare regulations. The methodological quality is concluded in **Table 6**.

**Table 6:** Risk of bias of the included animal studies.

Study	A	B	C	D	E	F	G	H	I	J	Total
Xu et al., 2011	+	?	+	?	?	+	+	?	?	+	5+
Wang et al., 2010	+	+	?	?	?	+	+	?	+	+	6+
Wang et al., 2013	+	+	?	?	?	+	+	?	+	+	6+
Song et al., 2013	+	+	+	?	?	+	+	?	?	+	6+
Xu et al., 2010	+	+	+	?	?	+	+	?	?	?	5+
Feng et al., 2010a	+	+	+	?	?	+	+	?	?	+	6+
Feng et al., 2009a	+	+	+	?	?	+	+	?	?	+	6+
Liu, 2016	–	+	+	?	?	+	+	?	?	+	4+
Feng et al., 2010b	+	+	+	?	?	+	+	?	?	+	6+
Wang et al., 2015	+	?	+	?	?	+	+	?	?	?	4+
Yao, 2016	+	?	+	?	?	+	+	?	?	?	4+
Liu et al., 2015	+	?	+	?	?	+	+	?	?	+	5+
Feng et al., 2010b	+	?	+	?	?	+	+	?	?	?	4+
Feng et al., 2010c	+	+	+	?	?	+	+	?	?	+	6+
Pan et al., 2010a	+	+	+	?	?	+	+	?	?	+	6+
Pan et al., 2010a	+	+	+	?	?	+	+	?	?	?	5+
Pan et al., 2009	+	+	+	?	?	+	+	?	?	+	6+
Li et al., 2009	+	?	+	?	?	+	+	?	?	+	5+
Feng et al., 2009c	+	+	+	?	?	+	+	?	?	+	6+
Pan et al., 2010b	+	+	+	?	?	+	+	?	?	+	6+
Bao et al., 2016	+	+	+	?	?	+	+	?	+	+	7+
Oh et al., 2015	+	+	+	?	?	+	+	?	+	+	7+
Qi et al., 2014	+	+	+	?	?	+	+	?	+	+	7+
Li et al., 2018	+	+	+	?	?	+	+	?	+	+	7+
Zhao et al., 2014	+	+	+	?	?	+	+	?	?	+	6+
Gao et al., 2011	+	+	+	?	?	+	+	?	?	+	6+
Ma et al., 2017	+	+	+	?	?	+	+	?	+	+	7+
Choi et al., 2011	+	+	?	?	?	+	+	?	+	+	6+
Hwang et al., 2014	+	+	+	?	?	+	+	?	+	+	7+
Shi et al., 2016	+	+	+	?	?	+	+	?	?	?	5+

Studies fulfilling the criteria of the following: (A) peer reviewed publication; (B) control of temperature; (C) random allocation to treatment or control; (D) blinded induction of model; (E) blinded assessment of outcome; (F) use of anesthetic without significant intrinsic neuroprotective activity; (G) animal model (aged or female involved); (H) sample size calculation; (I) compliance with animal welfare regulations; (J) statement of potential conflict of interests.

### Effectiveness

#### Randomized Control Trials

**Figure 2:**
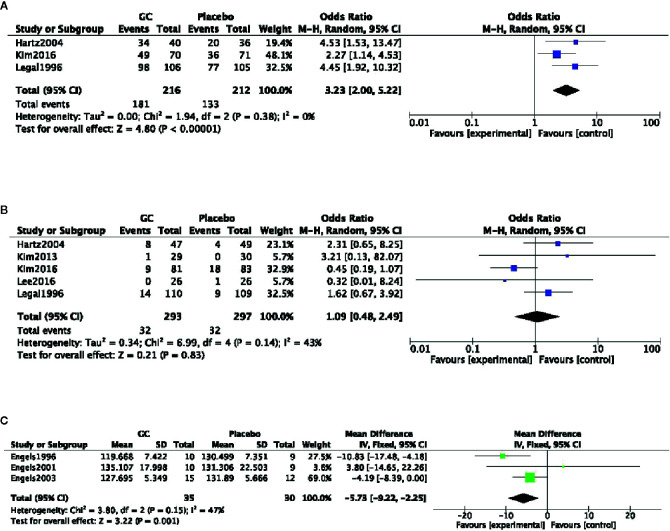
**(A)** Clinical effect of *Panax ginseng* C. A. Mey and its compounds versus Placebo. **(B)** Adverse events of *Panax ginseng* C. A. Mey and its compounds versus Placebo. **(C)** Heart rate recovery of *Panax ginseng* C. A. Mey and its compounds versus Placebo.

Five studies (Gal et al., 1996; Hartz et al., 2004; Hyeong-Geug et al., 2013; Kim et al., 2016; Lee et al., 2016) described the treatment of CFS patients. These studies utilized different scales of fatigue to measure therapeutic effect. Therefore, the meta-analysis was not conducted because of different evaluation criteria. All studies showed significant difference in their respective fatigue scales for PGC relative to placebo. Three studies (Gal et al., 1996; Hartz et al., 2004; Kim et al., 2016) reported clinical effect, which according to different self-reported fatigue scales. Meta-analysis of 3 studies (Gal et al., 1996; Hartz et al., 2004; Kim et al., 2016) found significant difference in clinical effect for PGC relative to placebo (nT/nC =181/133, OR=1.31, 95% CI: 1.17 to 1.46, P < 0 00001; heterogeneity χ^2^ = 1.94, df = 2 (P=0.38), I^2^ = 0%) (**Figure 2A**). Meta-analysis of 5 studies (Gal et al., 1996; Hartz et al., 2004; Hyeong-Geug et al., 2013; Kim et al., 2016; Lee et al., 2016) showed there was no significant difference in the frequency of adverse events between the groups (nT/nC =293/297, OR=1.09, 95% CI: 0.48 to 2.46, P=0.83, heterogeneity χ^2^ = 6.99, df = 4 (P=0.14), I^2^ = 0%) (**Figure 2B**).

Three studies (Engels et al., 1996; Engels et al., 2001; Engels et al., 2003) described the effect of PGC on physical performance recovery of healthy adults after exercise fatigue. Meta-analysis of 3 studies (Engels et al., 1996; Engels et al., 2001; Engels et al., 2003) found significant difference in heart rate recovery after exercise compared the PGC with placebo (nT/nC = 35/30; FE = −5.73; 95% CI, −9.22 to −2.55; *P* = 0.001; heterogeneity χ^2^ = 3.80, df = 2 (*P* = 0.15), *I* ^2^ = 47%) (**Figure 2C**).

#### Animal Studies

##### The Swimming Time

**Figure 3:**
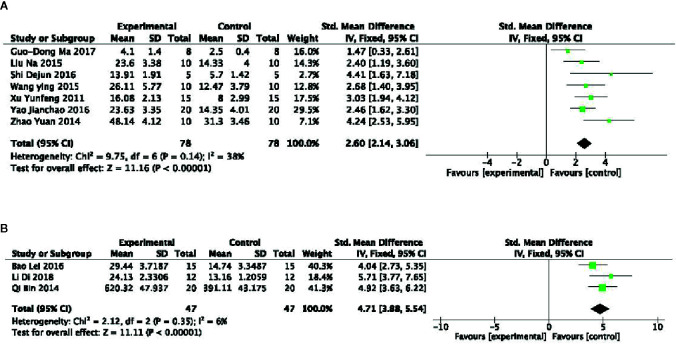
**(A)** Forced swimming test of *Panax ginseng* C. A. Mey and its compounds versus Control. **(B)** Weight-loaded swimming test of *Panax ginseng* C. A. Mey and its compounds versus Control.

Meta-analysis of 8 studies (Li et al., 2009; Xu et al., 2011; Zhao et al., 2014; Liu et al., 2015; Wang et al., 2015; Shi et al., 2016; Yao, 2016; Ma et al., 2017) showed PGC significantly increased the swimming time in WLST compared with control (n = 98, SMD =2.21, 95% CI: 1.83 to 2.59, P < 0 00001; heterogeneity: χ^2^ = 18.96, df = 7 (P =0.008), I^2^ = 63%). Owing to obvious heterogeneity, we used sensitivity analyses and removed one study (Li et al., 2009) that trained the mice before the swimming test. Meta-analysis of 7 studies showed a significant effect of PGC in increasing the swimming time in WLST compared with control (n = 78, SMD =2.60, 95% CI: 2.14 to 3.06, P < 0 00001; heterogeneity: χ^2^ = 9.75, df = 6 (P =0.14), I^2^ = 38%) (**Figure 3A**). Five studies (Choi et al., 2011; Song et al., 2013; Qi et al., 2014; Bao et al., 2016; Delgado et al., 2019) found that PGC was more effective in increasing the swimming time in FST than that of control (n = 67, SMD =2.78, 95% CI: 2.24 to 3.32, P < 0 00001; heterogeneity: χ^2^ = 41.15, df = 4 (P < 0 00001), I^2^ = 90%). Two studies (Choi et al., 2011; Song et al., 2013) may be considered as the probable source of the heterogeneity, in which 1 study (Song et al., 2013) utilized rats as experimental subjects and one study (Choi et al., 2011) utilized crude products of PG. After getting rid of these 2 studies, there was showing that PGC increasing the swimming time in FST (n = 47, SMD =4.71, 95% CI: 3.88 to 5.54, P < 0 00001; heterogeneity: χ^2^ = 2.12, df = 2 (P =0.35), I^2^ = 6%) (**Figure 3B**).

##### Serum Biochemical Parameters

**Figure 4:**
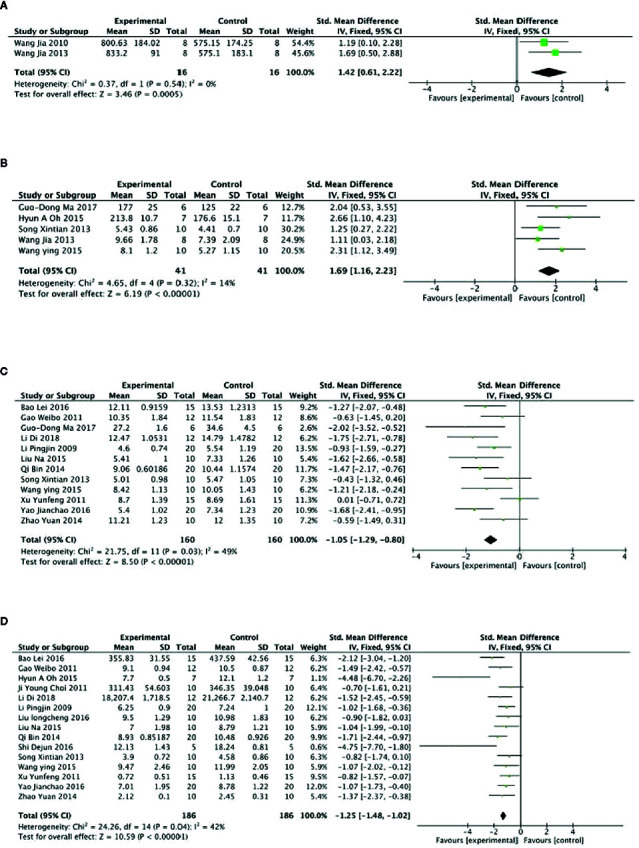
**(A)** Level of glutathione peroxidase in serum of *Panax ginseng* C. A. Mey and its compounds versus Control. **(B)** Level of glucose in serum of *Panax ginseng* C. A. Mey and its compounds versus Control. **(C)** Level of blood urea nitrogen in serum of *Panax ginseng* C. A. Mey and its compounds versus Control. **(D)** Level of blood lactate in serum of *Panax ginseng* C. A. Mey and its compounds versus Control.

**Figure 5:**
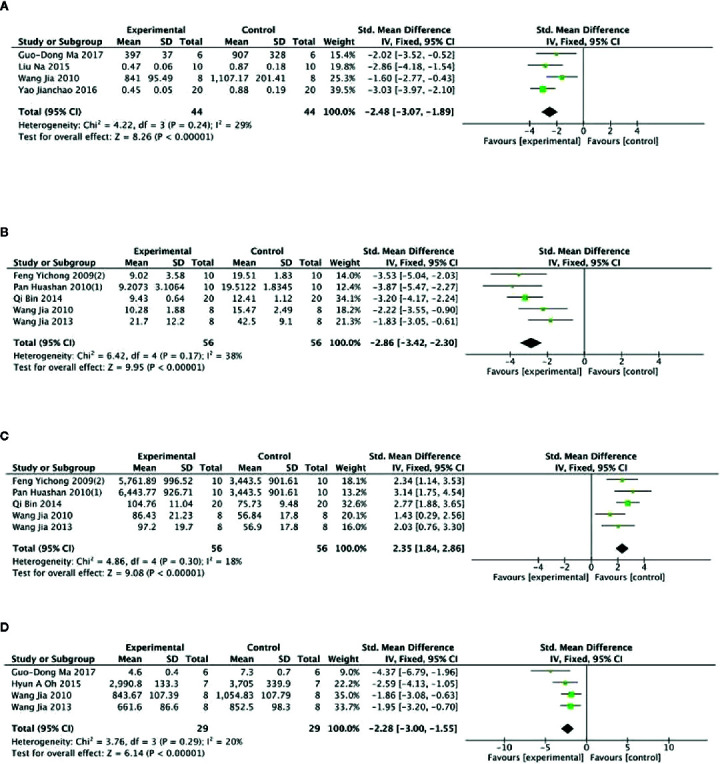
**(A)** Level of creatine kinase in serum of *Panax ginseng* C. A. Mey and its compounds versus Control. **(B)** Level of malondialdehyde in serum of *Panax ginseng* C. A. Mey and its compounds versus Control. **(C)** Level of superoxide dismutase in serum of *Panax ginseng* C. A. Mey and its compounds versus Control. **(D)** Level of lactic dehydrogenase in serum of *Panax ginseng* C. A. Mey and its compounds versus Control.

Meta-analysis of 3 studies (Qi et al., 2014; Wang et al., 2014; Zheng et al., 2017) showed PGC was significant for increasing level of GSH-Px compared with control (n = 47, SMD =2.16, 95% CI: 1.49 to 3.02, P < 0 00001; heterogeneity: χ^2^ = 46.87, df = 2 (P < 0 00001), I^2^ = 96%). After sensitivity analyses, we removed 1 study (Qi et al., 2014) that utilized ginseng protein as intervention. Meta-analysis of the rest of 2 studies found that PGC significantly increased the level of GSH-Px compared with control (n = 16, SMD =1.42, 95% CI: 0.61 to 2.22, P =0.0005; heterogeneity: χ^2^ = 0.37, df = 1 (P =0.54), I^2^ = 0%) (**Figure 4A**). Compared with controls, meta-analysis of 5 studies (Song et al., 2013; Wang et al., 2014; Wang et al., 2015; Oh et al., 2015; Ma et al., 2017) showed that PGC significantly increased level of GLU (n = 41, SMD =1.69, 95% CI: 1.16 to 2.23, P < 000001; heterogeneity: χ^2^ = 4.65, df = 4 (P =0.32), I^2^ = 14%) (**Figure 4B**); 11 studies (Li et al., 2009; Gao et al., 2011; Xu et al., 2011; Song et al., 2013; Qi et al., 2014; Zhao et al., 2014; Liu et al., 2015; Wang et al., 2015; Bao et al., 2016; Yao, 2016; Ma et al., 2017; Delgado et al., 2019) for reducing level of BUN (n = 160, SMD = −1.05; 95% CI, −1.29 to −0.80; *P* < 000001; heterogeneity: χ^2^ = 21.75, df = 10 (*P* < 0 00001), I^2^ = 49%) (**Figure 4C**); 15 studies (Li et al., 2009; Choi et al., 2011; Gao et al., 2011; Xu et al., 2011; Song et al., 2013; Qi et al., 2014; Zhao et al., 2014; Liu et al., 2015; Oh et al., 2015; Wang et al., 2015; Bao et al., 2016; Liu, 2016; Shi et al., 2016; Yao, 2016; Delgado et al., 2019) for reducing level of BLA (n = 186; SMD = −1.25; 95% CI, −1.48 to −1.02; P < 000001; heterogeneity: χ^2^ = 24.26; df = 14 (P =0.04); I^2^ = 42%) (**Figure 4D**); four studies (Liu et al., 2015; Yao, 2016; Ma et al., 2017; Zheng et al., 2017) for reducing level of CK (n = 44; SMD = −2.48; 95% CI, −3.07 to −1.89; P < 000001; heterogeneity: χ^2^ = 4.22, df = 3 (P=0.24), I^2^ = 29%) (**Figure 5A**); five studies (Feng et al., 2009c; Pan et al., 2010a; Qi et al., 2014; Wang et al., 2014; Zheng et al., 2017) for reducing level of MDA (n = 44; SMD = −2.86; 95% CI, −3.42 to −2.30; *P* < 0 00001; heterogeneity, χ^2^ = 6.42, df = 3 (*P* = 0.17), I^2^ = 38%) (**Figure 5B**); five studies (Feng et al., 2009c; Pan et al., 2010a; Qi et al., 2014; Wang et al., 2014; Zheng et al., 2017) for increasing level of SOD (n = 56; SMD = 2.35; 95% CI, 1.84 to 2.86, *P* < 000001; heterogeneity: χ^2^ = 4.86, df = 4 (P =0.30), I^2^ = 18%) (**Figure 5C**); four studies (Wang et al., 2014; Oh et al., 2015; Ma et al., 2017; Zheng et al., 2017) for reducing level of LDH (n = 29; SMD = −2.28; 95% CI, −3.00 to −1.55, *P* < 0 00001; heterogeneity: χ^2^ = 3.76, df = 3 (*P* = 0.29), I^2^ = 20%) (**Figure 5D**). Six studies (Li et al., 2009; Liu et al., 2015; Bao et al., 2016; Shi et al., 2016; Yao, 2016; Delgado et al., 2019) found that PGC was more effective for increasing activity of LDH than that of control (P< 0.05). No meta-analysis was carried out because of high heterogeneity.

##### Skeletal Muscle Biochemical Parameters:

**Figure 6:**
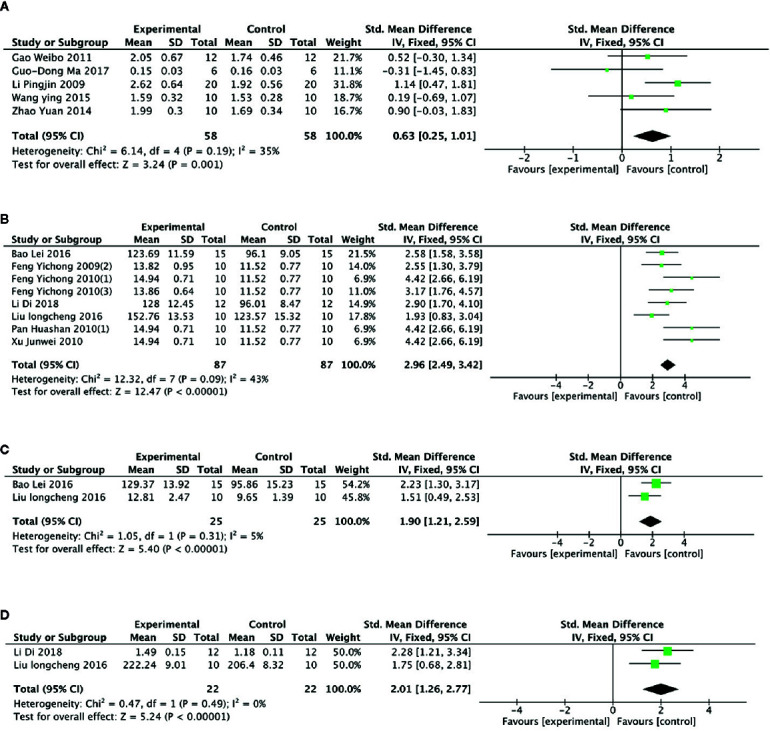
**(A)** Level of glycogen in muscle of *Panax ginseng* C. A. Mey and its compounds versus Control. **(B)** Activity of superoxide dismutase in muscle of *Panax ginseng* C. A. Mey and its compounds versus Control. **(C)** Activity of catalase in muscle of Panax ginseng C. A. Mey and its compounds versus Control. **(D)** Activity of glutathione peroxidase in muscle of *Panax ginseng* C. A. Mey and its compounds versus Control.

Meta- analysis of 6 studies (Li et al., 2009; Feng et al., 2010b; Gao et al., 2011; Zhao et al., 2014; Wang et al., 2015; Ma et al., 2017) showed a significant effect of PGC for increasing level of muscle glycogen compared with control (n = 68, SMD =0.80, 95% CI: 0.43 to 1.17, P < 000001; heterogeneity: χ^2^ = 17.10, df = 5 (P =0.004), I^2^ = 71%). After removing 1 studies (Feng et al., 2010b) that utilized rats as experimental subjects, meta- analysis of 5 studies (Li et al., 2009; Gao et al., 2011; Zhao et al., 2014; Wang et al., 2015; Ma et al., 2017) showed similar results (n = 58, SMD =0.63, 95% CI: 0.25 to 1.01, P =0.001; heterogeneity: χ^2^ = 6.14, df = 4 (P =0.19), I^2^ = 35%) (**Figure 6A**). Compared with control, meta-analysis of 9 studies (Feng et al., 2009c; Feng et al., 2010a; Feng et al., 2010b; Pan et al., 2010a; Pan et al., 2010b; Xu et al., 2010; Bao et al., 2016; Liu, 2016; Delgado et al., 2019) showed that PGC significantly improved activity of SOD (n = 97, SMD =2.61, 95% CI: 2.19 to 3.03, P < 0 00001; heterogeneity: χ^2^ = 23.71, df = 8 (P =0.003), I^2^ = 66%). Sensitivity analyses were conducted to explore potential sources of heterogeneity. After removing 1 trial (Pan et al., 2010b) that utilized crude products of PG, meta-analysis of 8 studies (Feng et al., 2009c; Feng et al., 2010a; Feng et al., 2010b; Pan et al., 2010a; Xu et al., 2010; Bao et al., 2016; Liu, 2016; Delgado et al., 2019) showed that PGC was still superior to the control (n = 87, SMD =2.96, 95% CI: 2.49 to 3.42, P < 000001; heterogeneity: χ^2^ = 12.32, df = 7 (P =0.09), I^2^ = 43%) (**Figure 6B**). Compared with control group, meta-analysis of 2 studies (Liu, 2016; Bao et al., 2016) showed that PGC had significant effect to increase activity of CAT (n = 25, SMD =1.90, 95% CI: 1.21 to 2.59, P < 0 00001; heterogeneity: χ^2^ = 1.05, df = 41 (P =0.31), I^2^ = 5%) (**Figure 6C**); 2 studies (Liu, 2016; Delgado et al., 2019) for improving activity of GSH-Px (n = 22, SMD =2.01, 95% CI: 1.26 to 2.77, P < 0 00001; heterogeneity: χ^2^ = 0.47, df = 1 (P =0.49), I^2^ = 0%) (**Figure 6D**). The level of MDA was reported in 9 studies (Feng et al., 2009c; Feng et al., 2010b; Feng et al., 2010c; Pan et al., 2010a; Pan et al., 2010b; Xu et al., 2010; Bao et al., 2016; Liu, 2016; Delgado et al., 2019). There was significant decreasing in the level of MDA compared PGC with control (P < 0.05). No meta-analysis was carried out because of high heterogeneity.

##### Liver Biochemical Parameters

**Figure 7:**
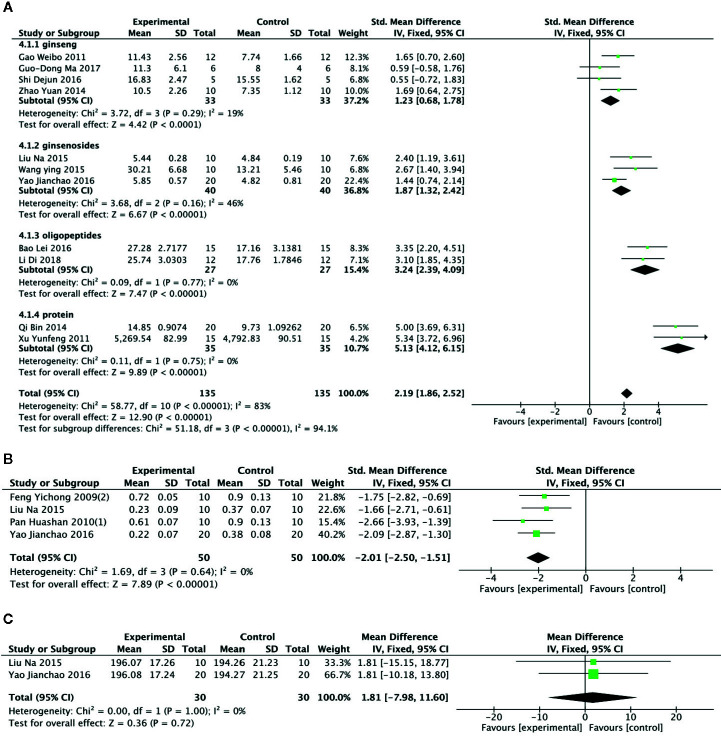
**(A)** Level of glycogen in Liver of *Panax ginseng* C. A. Mey and its compounds versus Control. **(B)** Activity of malondialdehyde in Liver of *Panax ginseng* C. A. Mey and its compounds versus Control. **(C)** Activity of glutathione peroxidase in Liver of *Panax ginseng* C. A. Mey and its compounds versus Control.

Meta-analysis of 11 studies (Gao et al., 2011; Xu et al., 2011; Zhao et al., 2014; Qi et al., 2014; Liu et al., 2015; Wang et al., 2015; Shi et al., 2016; Yao, 2016; Bao et al., 2016; Ma et al., 2017; Delgado et al., 2019) showed that PGC had significant effects for increasing level of hepatic glycogen compared with control (n = 135, SMD =2.19, 95% CI: 1.86 to 2.52, P < 000001; heterogeneity: χ^2^ = 58.77, df = 10 (P < 0 00001), I^2^ = 83%).Owing to obvious heterogeneity, we conducted subgroup analysis. These studies were divided into 4 subgroups according to different experiment interventions. Meta-analysis showed that there was significant difference in the level of glycogen between PGC and control in all subgroups, including ginseng (Gao et al., 2011; Zhao et al., 2014; Shi et al., 2016; Ma et al., 2017), Ginsenosides (Liu et al., 2015; Wang et al., 2015; Yao, 2016), ginseng protein (Xu et al., 2011; Qi et al., 2014), and ginseng oligopeptides (Bao et al., 2016; Delgado et al., 2019) groups (**Figure 7A**). Meta-analysis of 4 studies (Feng et al., 2009c; Pan et al., 2010a; Liu et al., 2015; Yao, 2016) showed that PGC significantly decreased level of MDA compared with control (n = 50; SMD = −2.01; 95% CI, −2.50 to −1.51; *P* < 000001; heterogeneity: χ^2^ = 1.69, df = 3 (*P* = 0.64), I^2^ = 0%) (**Figure 7B**). Meta-analysis of two studies (Liu et al., 2015; Yao, 2016) showed no significant difference in activity of GSH-Px between PGC and control (n = 30; SMD = 1.81; 95% CI, −7.98 to 11.60, *P* =0.72; heterogeneity: χ^2^ = 0.00, df = 1 (P =1.00), I^2^ = 0%) (**Figure 7C**). Activity of SOD was reported in four studies (Feng et al., 2009c; Pan et al., 2010a; Liu et al., 2015; Yao, 2016). Two studies (Feng et al., 2009c; Pan et al., 2010a) showed significant difference (*P* < 0.05) for increasing activity of SOD compared PGC with control, whereas 2 studies (Liu et al., 2015; Yao, 2016) showed contradictory results (*P* > 0.05).

##### Brain Tissue Biochemical Parameters

**Figure 8:**
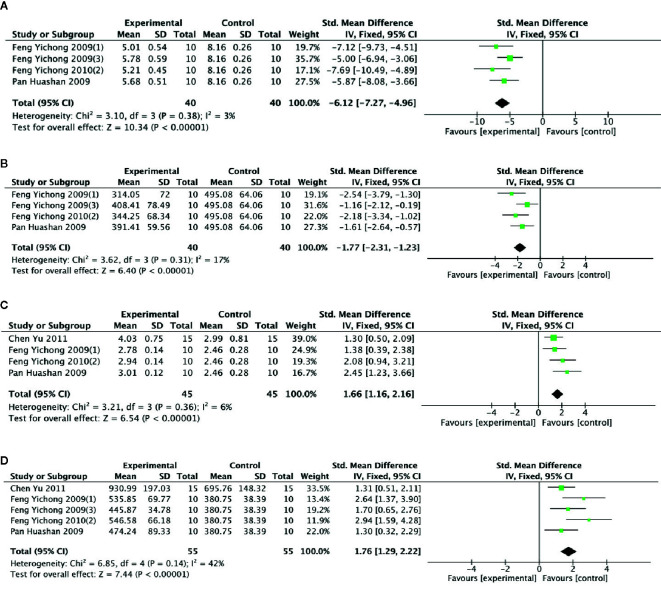
**(A)** Level of gamma-aminobutyric acid in brain tissue of *Panax ginseng* C. A. Mey and its compounds versus Control. **(B)** Level of 5-hydroxytryptamine in brain tissue of *Panax ginseng* C. A. Mey and its compounds versus Control. **(C)** Level of acetylcholine in brain tissue of *Panax ginseng* C. A. Mey and its compounds versus Control. **(D)** Level of dopamine in brain tissue of *Panax ginseng* C. A. Mey and its compounds versus Control.

Meta-analysis of 5 studies (Feng et al., 2009a; Feng et al., 2009b; Pan et al., 2009; Feng et al., 2010a; Chen and Li, 2011) showed PGC was significant for reducing level of GABA (n = 55; SMD = −4.45; 95% CI, −5.24 to −3.66; *P* < 0.00001; heterogeneity: χ^2^ = 17.97, df = 4 (P = 0.001), I^2^ = 78%) and level of 5-HT (n = 55; SMD = −1.19; 95% CI, −1.63 to −0.76; *P* < 0 00001; heterogeneity: χ^2^ = 15.52, df = 4 (P =0.004), I^2^ = 74%) compared with control. One study (Chen and Li, 2011) that utilize chloral hydrate to anesthetize rats may be considered as the probable source of the heterogeneity. After getting rid of this study, there was showing that PGC better reduce the level of GABA (n = 40; SMD = −6.12; 95% CI, −7.27 to −4.96; *P* < 000001; heterogeneity: χ^2^ = 3.10, df = 3 (*P* = 0.38), I^2^ = 3%) (**Figure 8A**) and 5-HT (n = 40; SMD = −1.77; 95% CI, −2.31 to −1.23; *P* < 0 00001; heterogeneity: χ^2^ = 3.62, df = 3 (*P* = 0.31), *I* ^2^ = 17%) (**Figure 8B**). Meta-analysis of five studies (Feng et al., 2009a; Feng et al., 2009b; Pan et al., 2009; Feng et al., 2010a; Chen and Li, 2011) found significant difference for increasing level of Ach between PGC and control groups (n = 55; SMD = 1.86; 95% CI, 1.38 to 2.23; *P* < 0 00001; heterogeneity: χ^2^ = 9.70, df = 4 (P =0.05), I^2^ = 59%). After removing one study (Feng et al., 2009a), meta-analysis of four studies (Feng et al., 2009b; Pan et al., 2009; Feng et al., 2010a; Chen and Li, 2011) showed similar results (n = 45; SMD = 1.66; 95% CI, 1.16 to 2.16, *P* < 000001; heterogeneity: χ^2^ = 3.21, df = 3 (*P* = 0.36), I^2^ = 6%) (**Figure 8C**). Meta-analysis of five studies (Feng et al., 2009a; Feng et al., 2009b; Pan et al., 2009; Feng et al., 2010a; Chen and Li, 2011) showed a significant increase in level of DA compared PGC with control (n = 55; SMD =1.76,; 95% CI, 1.26 to 2.22; *P* < 000001; heterogeneity: χ^2^ = 6.85, df = 4 (P = 0.14), I^2^ = 42%) (**Figure 8D**).

##### Animal Body Weight:

**Figure 9:**
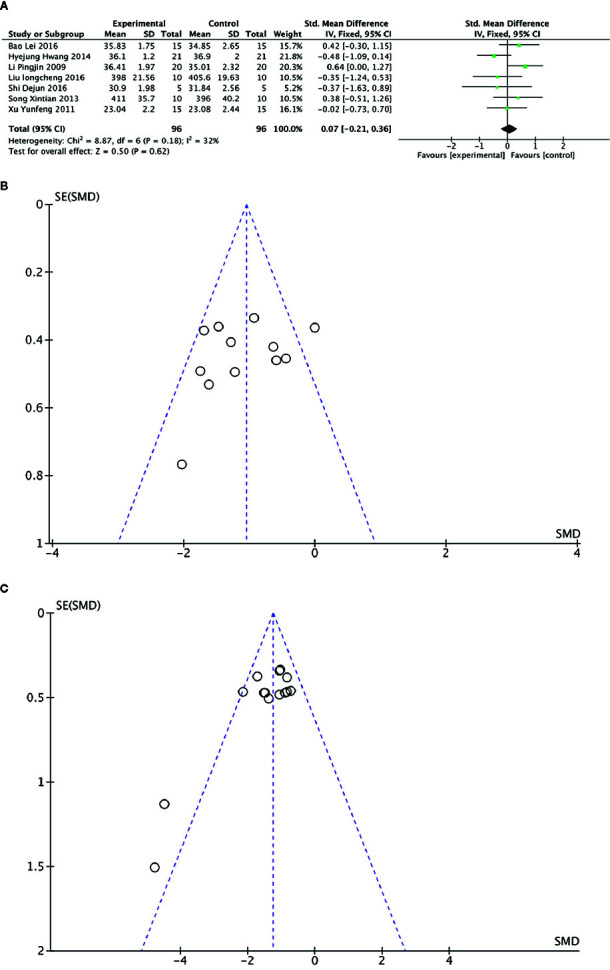
**(A)** Body weight of *Panax ginseng* C. A. Mey and its compounds versus Control. **(B)** Funnel plots of blood urea nitrogen. **(C)** Funnel plots of blood lactate.

Meta-analysis of 7 studies (Li et al., 2009; Xu et al., 2011; Song et al., 2013; Hwang et al., 2014; Bao et al., 2016; Liu, 2016; Shi et al., 2016) showed there was no significant difference in body weight between PGC and control groups (n = 96; SMD =0.07; 95% CI: −0.21 to 0.36; *P* = 0.62; heterogeneity: χ^2^ = 8.87, df = 6 (P = 0.18), I^2^ = 32%) (**Figure 9A**).

### Publication Bias

Funnel plots were conducted for two outcomes (**Figures 9B, C**). The results showed symmetrical distribution for the outcomes of BUN levels (Egger's test t = −1.05; 95% CI, −6.93 to 2.50; *P* = 0.320), which did not suggest an obvious publication bias. However, there was a significant bias in the BLA levels with Egger's test (*t* = −3.47; 95% CI, −5.14 to −1.19; *P* =0.004). Because the number of studies in the remaining outcomes was limited (n < 10), funnel plot and Egger's test were not appropriate.

### Possible Mechanisms

**Figure 10:**
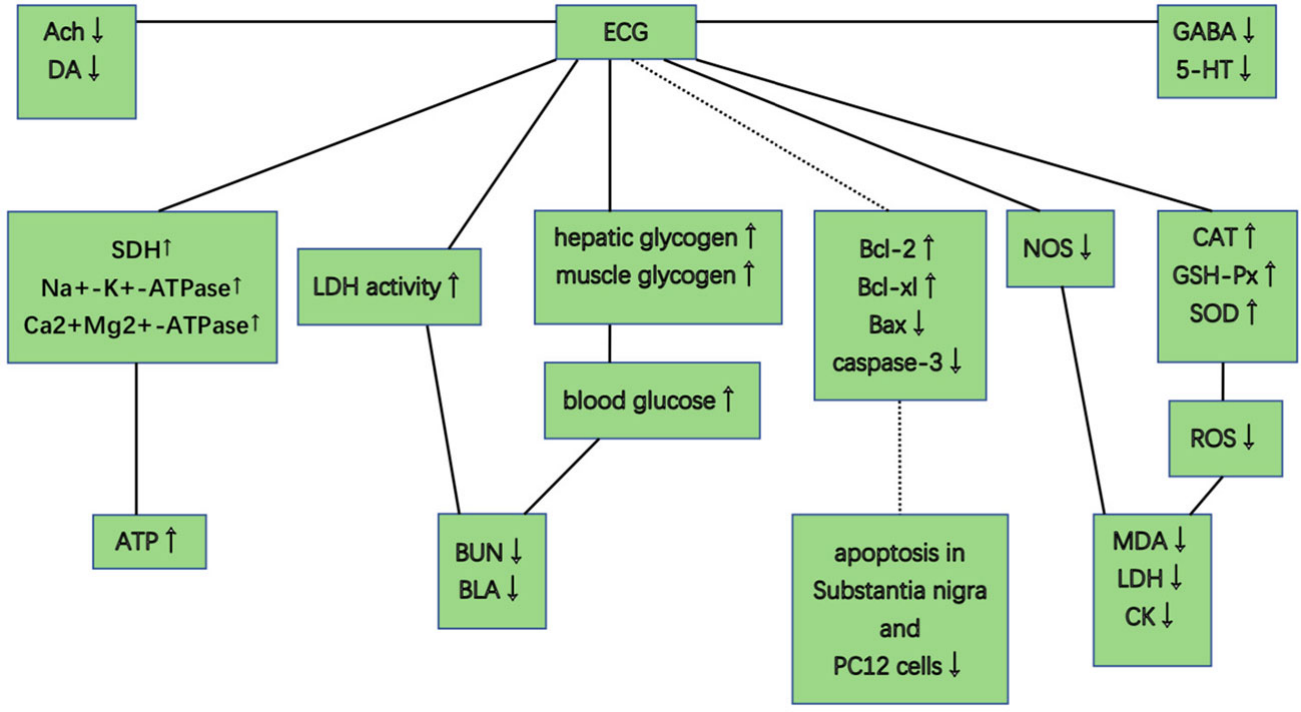
Possible mechanisms of *Panax ginseng* C. A. Mey and its compounds anti-fatigue.

PGC improved activity of GSH-Px, CAT, and SOD, scavenged free radicals and their metabolites, reduced the excessive ROS, and decreased levels of MDA, CK, and LDH. PGC decreased nitric oxide synthase (NOS), reduced toxic oxidant peroxynitrite, and prevented mitochondrial dysfunction and lipid peroxidation. PGC may enhance fat mobilization and promote gluconeogenesis, increase the delivery of glucose, and maintain blood glucose level. PGC increased the LDH activity and the hepatic glycogen levels, and retarded the accumulation of BUN and BLA. PGC improved succinate dehydrogenase (SDH), Na^+^-K^+^-ATPase, and Ca^2+^Mg^2+^-ATPase activities, enhanced mitochondrial function, and produced more adenosine triphosphate (ATP). PGC attenuated MPP^+^-induced MPTP-induced and apoptosis. PGC increased Ach and DA levels, and decreased GABA and 5-HT levels in brain tissue (**Figure 10**).

## Discussion

### Summary of Evidence

This is the clinical and preclinical systematic review to evaluate the efficacy and safety of PGC for fatigue. Eight RCTs with 678 participants and 30 studies with 2249 animals were selected. The quality of RCTs included was high, and animal studies were generally moderate. The findings of RCTs demonstrated that PGC was superior to placebo according to their respective fatigue scales, heart rate recovery, and clinical effect. There were a similar number of adverse effects between PGC and placebo group. The evidence available from animal studies showed that PGC could preserve physical function after exercise, mainly through antioxidant, regulating carbohydrate metabolism, delaying the accumulation of metabolites, promoting mitochondrial function, neuroprotection, antiapoptosis, and regulating neurotransmitter disorder in central nervous system.

### Limitations

Some methodological flaws existed in the primary RCTs. First of all, 3 studies used allocation concealment (Hartz et al., 2004; Hyeong-Geug et al., 2013; Lee et al., 2016). Trials with unreported or inadequate allocation concealment could be exaggerated an average 18% beneficial effect of interventions (Higgins and Green, 2011). Second, non-blinding of outcome assessment may lead to systemic errors. In previous reviews, non-blinding of outcome assessment can lead to an overestimation of treatment effect by 27% to 68%, depending on different outcome types, i.e., binary outcome, measurement scale outcome, and time-to-event outcome. However, in RCTs, blinded outcome assessment was commonly poor reported and used. In present study, only 2 RCTs (Hartz et al., 2004; Lee et al., 2016) reported blinding of outcome assessment. The observer bias can be avoided by sufficient blinding. More independent assessors can be further used to increase the feasibility of blind assessment (Brennan et al., 2015). Thirdly, 3 RCTs (Hyeong-Geug et al., 2013; Kim et al., 2016; Lee et al., 2016) formally registered. Clinical trial registration could help to minimize bias in selective reporting and improve the validity and value of the scientific evidence (Angelis et al., 2006). Fourthly, intention to treat (ITT) analysis is a strategy to gather data as completely as possible on all randomized patients in line with their scheduled assessments (Lewis and Machin, 1993). Four RCTs (Hermann-J et al., 2003; Hyeong-Geug et al., 2013; Kim et al., 2016; Lee et al., 2016) described whether they analyzed the data based on the ITT principle. Trials with incorrect or no ITT analysis may overestimate the results (Bondemark and Abdulraheem, 2017). Finally, various scales of fatigue were used as outcome measure in different trials. The evaluation of clinical efficacy rate is based on inconsistent scales, which restrict the validity and reliability. The quality of animal studies was considered to be moderate, suggesting that the results should be carefully interpreted. Fatigue can be divided into CFS and post-exercise fatigue in clinic. In the present analysis, all fatigue models are exhaustion, which may lead to ignore chronic process.

In addition, we cannot neglect the contribution of Korea and other countries worldwide in the study of ginseng in treatment of fatigue. Due to the limitation of language, the present study was not included studies which language was not English or Chinese. We can increase international cooperation to overcome the linguistic limitation.

### Implications

The evidence available from present study supported the routine use of PGC for fatigue, whereas the safety still needs more data because only five of eight studies reported. Given the gap between limitations of the primary RCTs and the quality of RCTs, we recommend that further design of the RCTs should consult the CONSORT statement (Moher et al., 2005), which offer a standard way for authors to design, conduct, analyze, and interpret, and to assess the validity of results.

Currently, there is no gold standard for measuring fatigue. Various scales were used to measure fatigue in different studies. Some scales have been calibrated, and some are homemade. Measurement of fatigue is challenging. Due to the wide range of conceptualizations of the problem and the concurrent development of questionnaires for many specific diseases, many questionnaires are used to measure fatigue. A comprehensive fatigue measurement, such as the Fatigue Severity Scale (Krupp et al., 1989), Piper Fatigue Scale (Piper et al., 1998), or FACIT-F (David et al., 2010), assesses the impact of fatigue on daily activities and its severity. In addition, short fatigue measurements such as the POMS-B fatigue subscale (Mcnairpm and Dropplemannl, 1992) and the 7-item Patient Reported Outcome Measurement Information System Cancer Fatigue Short Form (Cessna et al., 2016; Garcia et al., 2016) mainly assess severity of fatigue. The fatigue measurement precision with a comprehensive measure was greater than that with short fatigue measurement in evaluating moderate to severe fatigue, whereas the short fatigue measurement performed better in evaluating mild fatigue (Voshaar et al., 2015). Therefore, we should select the suitable fatigue measurements based on the research requirements.

Degree and scope of debilitating fatigue is a core component of health care where chronic diseases are receiving increasing attention. Current acute disease research models are not enough to solve chronic disorders affecting multiple regulatory systems and present complex constellations of symptoms. The identification of objective markers consistently associated with CFS is an important goal in relation to diagnosis and treatment, as the current case definitions are based entirely on physical signs and symptoms. Since the human body is an autonomous, fully integrated, and self-regulating system, it is not surprising that even localized muscle fatigue can present systemic biomarkers. There is a growing study devoted to understanding the biology of fatigue. Recognition of CFS biomarkers is an important part of this work. A complex construct of symptoms emerges from alterations and/or dysfunctions in the nervous, endocrine, and immune systems. Biomarkers, distributing across these systems, constituted complex biological networks. The acquisition of biomarkers required a comprehensive biological network-based analysis of fatigue biology (Klimas et al., 2012). In addition, molecular aberrations observed in many CFS blood cell studies provided an opportunity to develop diagnostic analysis of blood samples. With the development of micro/nanofabrication, direct electrical detection of cellular and molecular properties, microfluidics, and artificial intelligence techniques, a nano-electronics blood-based assay have been developed, which can potentially establish diagnostic biomarkers and drug screening platform for CFS (Esfandyarpour et al., 2019).

Preclinical research is the key to convert preclinical data into clinical data (Ramirez et al., 2017). However, there is growing concern that poor experimental design and transparent reporting lead to frequent failure of translating preclinical discoveries into new therapies for human diseases (Hackam and Redelmeier, 2006). In present study, the quality of including animal studies was moderate. We recommend that further design of the studies should consult the ARRIVE guidelines (Kilkenny et al., 2012) and use appropriate animals, random allocation, model blinded induction, and outcomes blinded assessment to improve the accuracy of the results.

PGC acted through complex, multicompound, multitarget, and multipathway mechanisms in fatigue and might prove to be of great value in further clinical trials. The possible mechanisms of PGC for fatigue are summarized as follows: (1) Antioxidant stress: PGC passed through the injured membrane, improved the activity of GSH-Px, CAT, and SOD, scavenged hydroxyl radical, and reduced the excessive ROS, and thus preventing lipid oxidation and protecting the corpuscular membrane to reduce the release of LDH, MDA, and CK into the serum (Wang et al., 2014; Zheng et al., 2017). Another mechanism might involve the nitric oxide pathway. NOS, a pro-oxidative enzyme, increased the production of toxic oxidant peroxynitrite. PGC decreased NOS and prevented peroxynitrite-induced mitochondrial dysfunction and lipid peroxidation (Ki Sung et al., 2006); (2) Regulation of carbohydrate metabolism: PGC increased the proportion of energy supplied by fat and promoted gluconeogenesis to improve hepatic glycogen storage. PGC enhanced the delivery of glucose by increasing capillary perfusion and plasma glucose concentration and increased the permeability of the muscle membrane of glucose to increase the muscle glucose uptake during exercise (Wang et al., 2015; Ma et al., 2017). A reduced rate of hepatic and muscle glycogen break-down and a greater potential for fatty acid metabolism could maintain blood glucose level, and thus enhancing exercise capacity (Favier and Koubi, 1988); (3) Delaying the accumulation of metabolites: With the accumulation of BUN and BLA, the pH in muscle tissue and blood reduced, which could obstruct the transmission of excitation at neuromuscular junctions, reduce the maximum tension and sustainability of muscle tissue, and hinder the process of sugar supply. PGC increased the LDH activity and the glycogen levels and retarded the accumulation of BUN and BLA (Oh et al., 2015; Delgado et al., 2019); (4) Promotion of mitochondrial function: SDH is a key enzyme associated with the regulation of the tricarboxylic acid cycle, catalyzing the synthesis of ATP. In addition, Na^+^-K^+^-ATPase and Ca^2+^-Mg^2+^-ATPase are crucial enzymes to degrade ATP. NRF-1 and TFAM are positive regulators of transcription. PGC improved SDH, Na^+^-K^+^-ATPase, and Ca^2+^Mg^2+^-ATPase activities. PGC enhanced the mRNA expression of NRF-1 and TFAM and increased the mtDNA content, and thereby enhancing mitochondrial function and producing more ATP for energy supplementation (Delgado et al., 2019); (5) Neuroprotection and antiapoptosis: PGC had a protective effect against MPTP-induced apoptosis in the mouse substantial nigra. This anti-apoptotic effect of PGC may be attributed to enhanced expression of Bcl-2 and Bcl-xl, reduced expression of bax and nitric oxide synthase, and inhibited activation of caspase-3 (Radad et al., 2006); (6) Regulation of neurotransmitter disorder: PGC decreased GABA and 5-HT levels, thereby increasing central nervous system excitability. PGC decreased the activity of acetylcholinesterase, maintained normal Ach and norepinephrine levels in cholinergic neurons, and enhanced the level of DA in the hippocampus (Feng et al., 2009b; Chen and Li, 2011).

## Conclusions

The findings of present study demonstrated that PGC exerted anti-fatigue function, mainly through antioxidant stress, regulation of carbohydrate metabolism, delaying the accumulation of metabolites, promotion of mitochondrial function, neuroprotection and antiapoptosis, and regulation of neurotransmitter disorder. And the findings supported, at least to an extent, the use of PGC for fatigue.

## Author Contributions

Study conception and design: YL/G-QZ. Acquisition, analysis and/or interpretation of data: T-YJ/P-QR/H-YL/P-PZ/G-QZ/YL Final approval and overall responsibility for this published work: YL/G-QZ.

## Funding

This study was supported by a grant from the National Natural Science Foundation of China (81973657/H2902); priority speciality of integrative brain diseases, the second affiliated hospital of Wenzhou Medical University, Wenzhou China (2016).

## Conflict of Interest

The authors declare that the research was conducted in the absence of any commercial or financial relationships that could be construed as a potential conflict of interest.
